# Lowering the affinity of single-chain monovalent BBB shuttle scFc-scFv8D3 prolongs its half-life and increases brain concentration

**DOI:** 10.1016/j.neurot.2024.e00492

**Published:** 2024-12-04

**Authors:** Andrés de la Rosa, Nicole G. Metzendorf, Jonathan Efverström, Ana Godec, Dag Sehlin, Jamie Morrison, Greta Hultqvist

**Affiliations:** a Department of Pharmacy, Uppsala University, Uppsala, Sweden; b Department of Public Health and Caring Sciences, Uppsala University, Uppsala, Sweden

**Keywords:** Blood brain barrier, Transporter, Antibodies, Affinity, Monovalent

## Abstract

Monoclonal antibody therapeutics is a massively growing field. Progress in providing monoclonal antibody therapeutics to treat brain disorders is complicated, due to the impermeability of the blood-brain barrier (BBB) to large macromolecular structures. To date, the most successful approach for delivering antibody therapeutics to the brain is by targeting the transferrin receptor (TfR) using anti-TfR BBB shuttles, with the 8D3 antibody being one of the most extensively studied in the field. The strategy of fine-tuning TfR binding affinity has shown promise, with previous results showing an improved brain delivery of bivalent 8D3-BBB constructs. In the current study, a fine-tuning TfR affinity strategy has been employed to improve single-chain variable fragment (scFv) 8D3 (scFv8D3) affinity mutants. Initially, *in silico* protein-protein docking analysis was performed to identify amino acids (AAs) likely to contribute to 8D3s TfR binding affinity. Mutating the identified AAs resulted in decreased TfR binding affinity, increased blood half-life and increased brain concentration. As monovalent BBB shuttles are seemingly superior for delivering antibodies at therapeutically relevant doses, our findings and approach may be relevant for optimizing brain delivery.

## Introduction

The field of monoclonal antibody therapeutics is rapidly growing and currently there are nearly 1200 antibody therapeutics in clinical trials [1] and very many already approved. However, hardly any of these have a target in the brain due to the challenge of effectively crossing the blood-brain barrier (BBB) [2,3]. The BBB protects the brain by strictly regulating substances that can reach the brain from the blood circulation [4]. This makes the delivery of antibody therapeutics into the brain very difficult. Only 0.1 ​% of intravenously injected antibodies reach the cerebrospinal fluid [5] and even less reach the brain parenchyma (0.009 ​± ​0.001 ​%) [6]. The most successful strategy to transport antibodies across the BBB has been to utilize one of the inherent mechanisms capable of transporting macromolecules into the brain, referred to as receptor-mediated transcytosis (RMT). In RMT, antibodies equipped with a dedicated receptor-binding domain can utilize this pathway by binding such receptors on the apical endothelial cell (EC) surface of the BBB. After binding to the receptor, the antibody-receptor complex is internalized into the cell by endocytosis, transported through the cell by intracellular vesicular trafficking known as transcytosis and finally released into the brain parenchyma by exocytosis at the basolateral side of the EC surface [7]. Presently, targeting the transferrin receptor (TfR) has generated the most promising results for brain delivery of antibodies [[8], [9], [10]] including one in clinical trials (add ref https://www.ncbi.nlm.nih.gov/pmc/articles/PMC10572082/). These antibodies can be designed to bind to TfR with one or more domains [[11], [12], [13], [14], [15], [16], [17], [18], [19], [20], [21], [22]]. 8D3 [23,24] is a high affinity antibody that binds to an epitope on the extracellular apical domain of the murine TfR (mTfR), distinct to that of the endogenous ligand transferrin [8]. 8D3 has been used extensively in different formats, in our lab [13,22,25,26] and by others [16,[27], [28], [29], [30]], for brain-delivery across the BBB. Recent studies have reported that fine-tuning the mTfR-affinity of a bivalently binding 8D3-containing antibody construct, by moderately lowering its affinity, improved its brain concentration [31].

In addition to affinity, TfR binding valency is an important factor determining the degree of transcytosis antibodies can achieve [8], with evidence suggesting that monovalent binding is superior for brain uptake compared to bivalent binding [14]. Bivalent binders have been proposed to crosslink TfR receptors on the cell surface, promoting sorting of endocytosed antibody-receptor complexes to lysosomal degradation instead of transcytosis [14,32]. The likelihood of crosslinking increases with higher concentration of bivalent TfR binders [33]. Furthermore, due to the avidity effect [34], which is the binding with multiple domains simultaneously, bivalent binders have slower TfR dissociation rates, thereby resulting in increased affinity to TfR [19,21,35]. Too slow TfR dissociation rate and too high affinity have also been proposed to promote lysosomal degradation, causing TfR down-regulation [14,[35], [36], [37]]. However, it is experimentally difficult to distinguish this from crosslinking for bivalent binders. Earlier studies have shown that a high affinity monovalent TfR binder, that bind in a different way than 8D3, co-localize with lysosomal markers *in vitro* and cause degradation of cortical TfR *in vivo* [35]. Lowering the TfR binding affinity of this binder resolved these issues, resulting in greater brain delivery, but also prolonged blood half-life [31,32,37,38]. Prolonging half-life can lead to increased brain exposure over time. It is difficult to distinguish whether the increased brain delivery observed for monovalent low-to-moderate TfR affinity binders [35,37,38] is due to increased TfR transcytosis and/or prolonged half-life. Results from in vitro BBB models show that the results are also translatable to the humanTfR [39].

In this study, we initially used *in silico* protein-protein docking tools to design scFv8D3 affinity mutants, which were recombinantly conjugated to an in-house designed single-chain Fc antibody domain (scFc-scFv8D3), providing monovalent TfR binding constructs with variable affinities which have not been studied before [40]. We subsequently analyzed these monovalent 8D3 TfR binding affinity mutants using a combined in-house derived *in vitro* transcytosis assay (In-Cell BBB-Trans assay) [40] together with *in vivo* studies, to discriminate the effect of transcytosis from the effect of increasing brain exposure over time. We show that fine-tuning the affinity of a monovalent scFv8D3 by introducing point mutations targeting amino acids in the paratope of scFv8D3, resulted in lowered TfR binding affinity, longer blood half-life and significantly higher brain concentration compared to the wildtype TfR binder. The results of this study provide a simplified experimental approach and rationale for exploring the possibility of improving brain uptake of monoclonal antibody therapeutics to the brain through fine-tuning TfR binding affinities.

## Results

### Identifying amino acids potentially influencing the mTfR affinity of scFv8D3

**Fig. 1:**
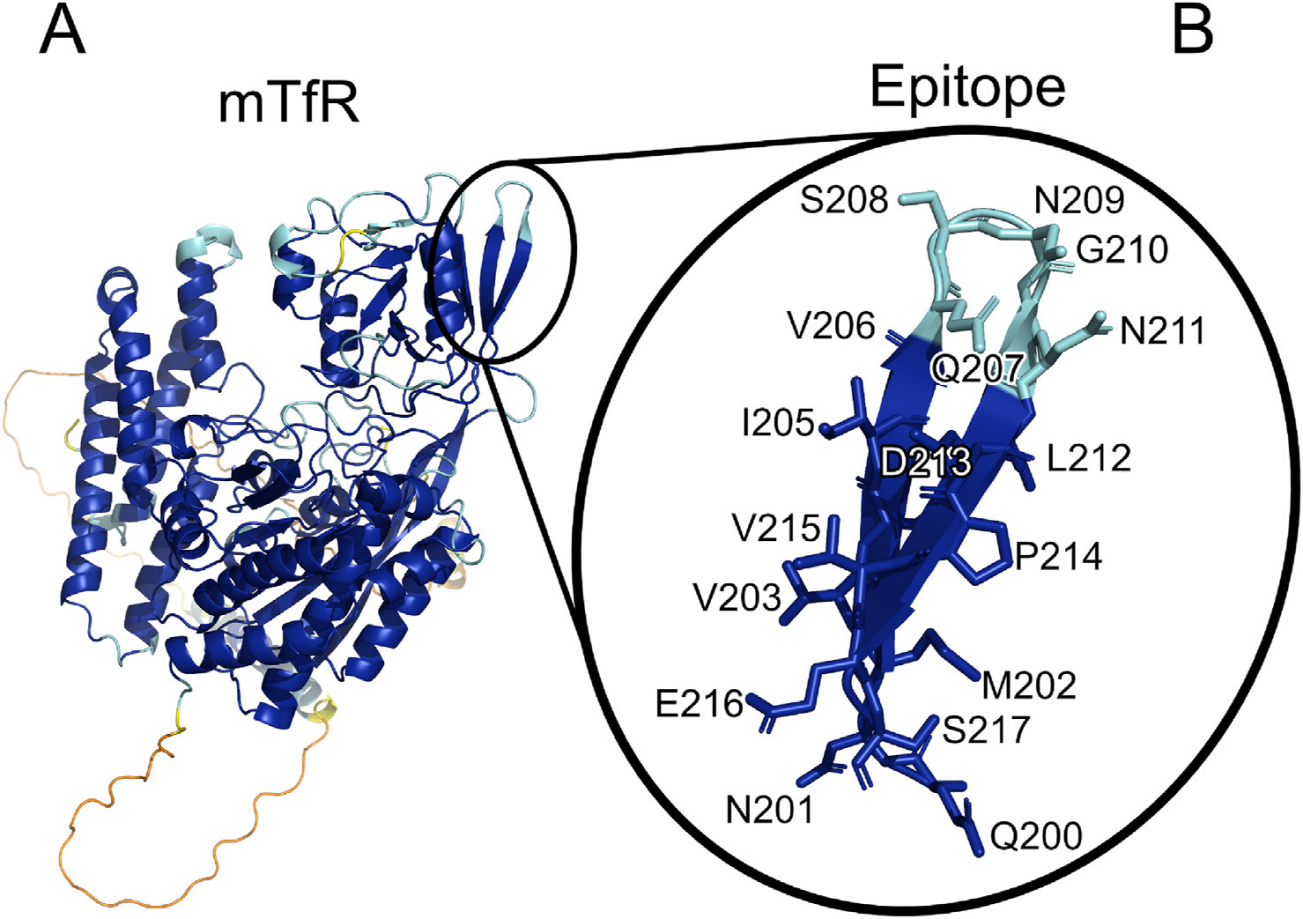
mTfR and the epitope of scFv8D3. (A). mTfR modeled by AlphaFold2 [45]. (B). Close-up of scFv8D3 mTfR epitope depicted with stick representation. Colored according to AlphaFold2s predicted local distance difference test (pLDDT) score, which represents the accuracy of the model; blue ​= ​pLDDT >90, turquoise ​= ​90 ​> ​pLDDT >70, yellow ​= ​70 ​> ​pLDDT >50, orange ​= ​pLDDT <50.

To create point mutants of scFv8D3 with decreased mTfR affinity, the amino acids (AA)s of scFv8D3 and its mTfR epitope were analyzed to identify targets potentially influencing mTfR affinity (Fig. 1A and B). Out of the three complementarity-determining regions (CDRs) of antibodies, the third CDR in the heavy chain (VHCDR3) is most often the region that primarily determines antibody-antigen specificity and affinity [[41], [42], [43], [44]]. Therefore, the AAs in the VHCDR3 region of scFv8D3 and the AAs of the mTfR epitope were analyzed based on the properties of their respective functional groups, referred to as AA functional group analysis.

### Amino acid functional group analysis

**Fig. 2:**
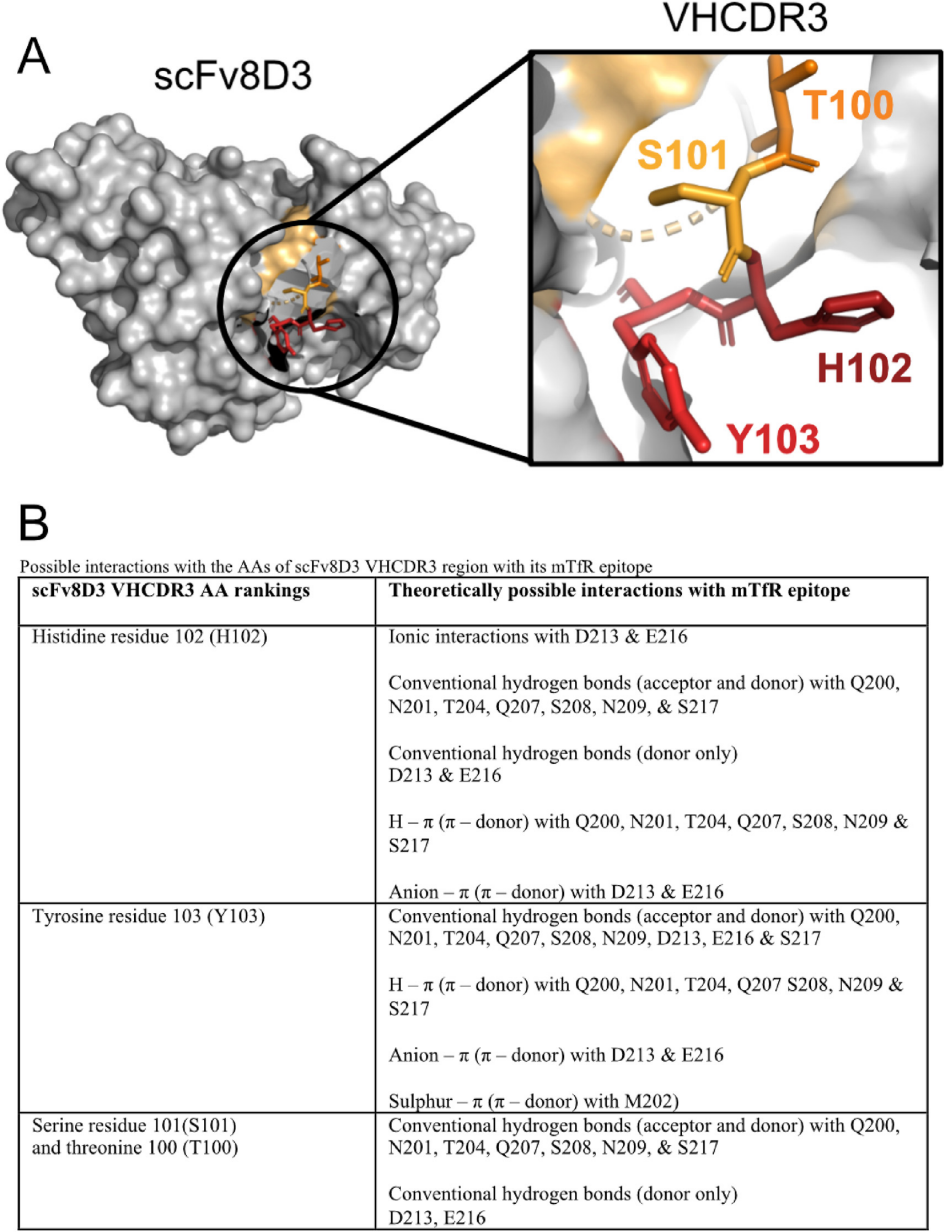
A. Model of scFv8D3 highlighting AAs within the VHCDR3-region with the highest number of potential interactions with its mTfR epitope. Ranking of the AAs is illustrated by shade where the darkest shade represents the highest ranking. B. Ranking and summary of potential interactions between the VHCDR3 AAs of scFv8D3 and its epitope on mTfR.

To identify the AAs in the VHCDR3 region with the highest potential to form strong interactions with the mTfR epitope, all theoretically possible strong interactions between the AAs present within the respective regions were identified. The possible interactions looked for were ionic interactions [46], hydrogen bonding, conventional and hydrogen – π, (H– π) [[46], [47], [48]], π – π interactions [46,48,49], cation – π interactions [46,48,50,51], anion – π interactions [46,48,[52], [53], [54]] and sulfur – π interactions [46,48,55,56]. This work was done manually without the use of any software. The AAs in the VHCDR3 region were ranked from highest to lowest likelihood of participating in strong interactions in the following order: histidine residue at position 102 (H102), tyrosine residue 103 (Y103), with serine residue 101 (S101) and threonine 100 (T100) at a shared third place (Fig. 2A). Their rankings and potential interactions are summarized in (Fig. 2B).

### In silico protein-protein docking analysis

To complement the result of the AA functional group analysis, *in silico* protein-protein docking analysis was performed. The results generated were considered rough predictions of potential interactions and therefore, the analysis was only considered in relation to the antecedent AA functional group-analysis. The protein-protein docking was performed with two different softwares, ezPPDock and AbAdapt, to increase the reliability of the results. In short, a homology-modeled structure of scFv8D3 was used together with a shortened mTfR peptide retaining the secondary structure of the epitope, modeled using AlphaFold2 (Supplementary Fig. 1B). This shortened mTfR peptide was used instead of the whole mTfR receptor to facilitate the protein-protein docking simulations and to further refine the predictions generated. Restrictions were imposed by blocking interactions outside of the mTfR epitope. To rank the individual AAs of the VHCDR3 region of scFv8D3 in terms of their probability of epitope-interaction, out of the 2001 generated predictions 15 were selected after visual inspection with the inclusion criteria of having at least one AA of the scFv8D3 VHCDR3 within 4.5 ​Å interaction distance of the mTfR epitope. The number of positions in which the AAs were within interaction distance of the mTfR epitope were counted and their observed frequencies are shown in Table 1. In addition, since more than one of the VHCDR3 AAs may be within interaction distance of the epitope at the same time their frequency are also shown as percentage of occurrences within the selected 15 predictes poses of ezPPDock and for AbAdapt the same was done for 30 of the first 31 poses generated. These 30 poses were selected based on having the recommended score of ≥20. The ranking of the AAs in the protein-protein docking analysis resulted in the following ranking: H102, Y103, followed by serine residue 101 (S101) and threonine 100 (T100) at a shared third place (Table 1), which supported the conclusion of the AA functional group analysis.

**Table 1:** AAs of scFv8D3 VHCDR3 region ranked according to the number of protein-protein docking poses within interaction distance of its mTfR epitope.

Top ranked AAs in scFv8D3 VHCDR3	Number of poses within interaction distance of epitope		Percentual presence of AA within interaction distance of epitope in protein-protein docking poses (%)	
	ezPPDock software	AbAdapt software	ezPPDock	AbAdapt
H102	9	17	60.0	56.6
Y103	8	5	53.3	16.7
T100	0	3	0.00	10.0
S101	2	0	13.3	0.00

### Choosing amino acid targets to create scFv8D3 affinity mutant constructs

**Fig. 3:**
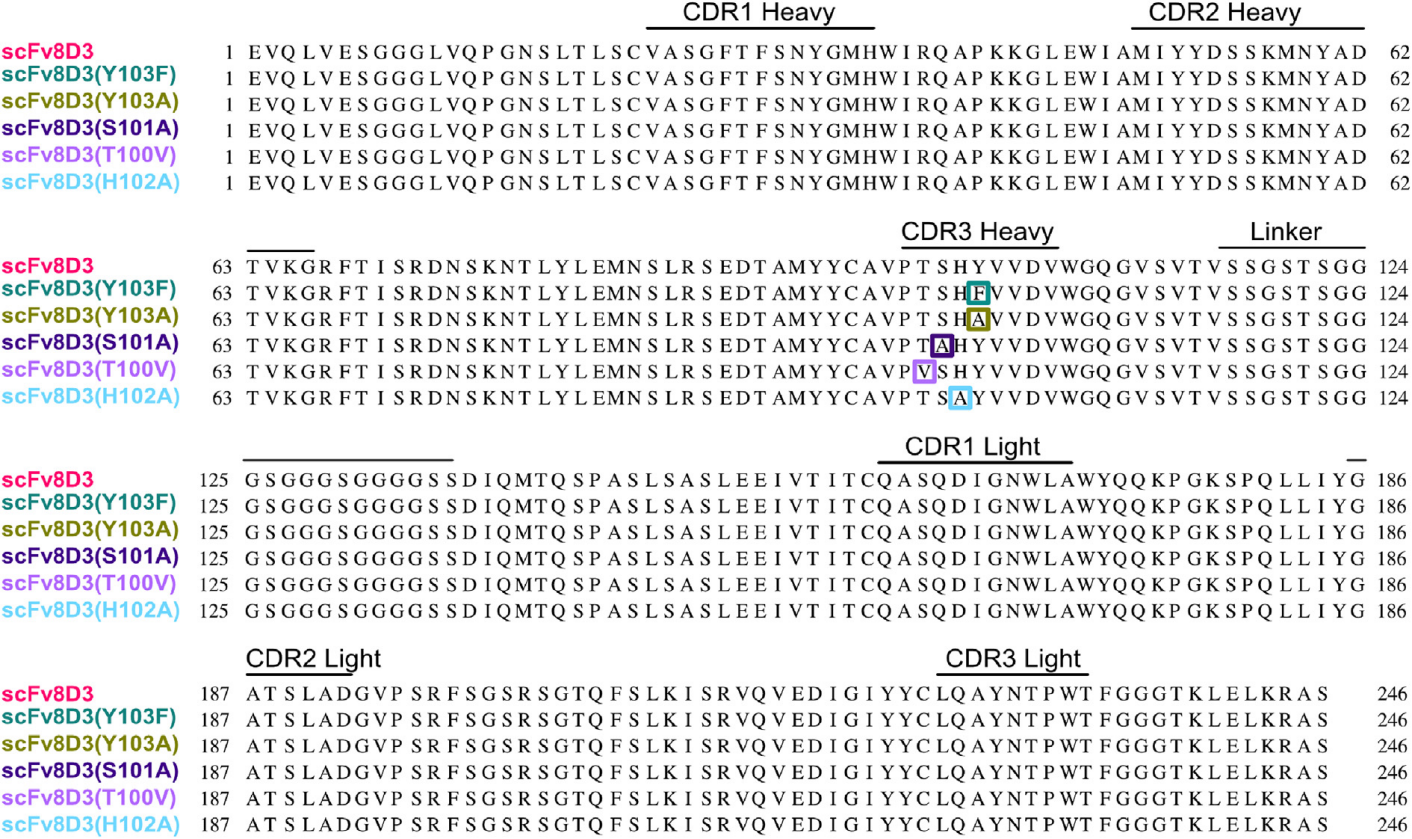
scFv8D3 and the point-mutated scFv8D3 sequences alignment. The AA sequences of scFv8D3 and the point-mutated scFv8D3 sequences pertaining to the affinity mutant constructs scFc-scFv8D3(Y103F), scFc-scFv8D3(Y103A), scFc-scFv8D3(S101A), scFc-scFv8D3(T100V), and scFc-scFv8D3(H102A) aligned. The single point mutations introduced in the VHCDR3 region of the affinity mutant constructs are highlighted in their respective colors.

When choosing the AAs to target to create point-mutants of scFv8D3 with decreased TfR binding strength, the results from the AA functional group analysis and the *in silico* protein-protein docking analysis were considered. Point mutations were made targeting the AAs identified as potentially influencing the TfR affinity of scFv8D3, creating five affinity mutant constructs (Fig. 3): scFc-scFv8D3(T100V), scFc-scFv8D3(S101A), scFc-scFv8D3(H102A), scFc-scFv8D3(Y103A) and scFc-scFv8D3(Y103F). To limit the risk of decreasing the affinity too much by conventional alanine mutations [41], the scFc-scFv8D3(T100V) and scFc-scFv8D3(Y103F) mutants were designed to retain part of their functional group properties as in both cases only the OH-groups were removed by the mutations. For T100 the OH-group was replaced with a methyl group.

### Production of constructs

**Fig. 4:**
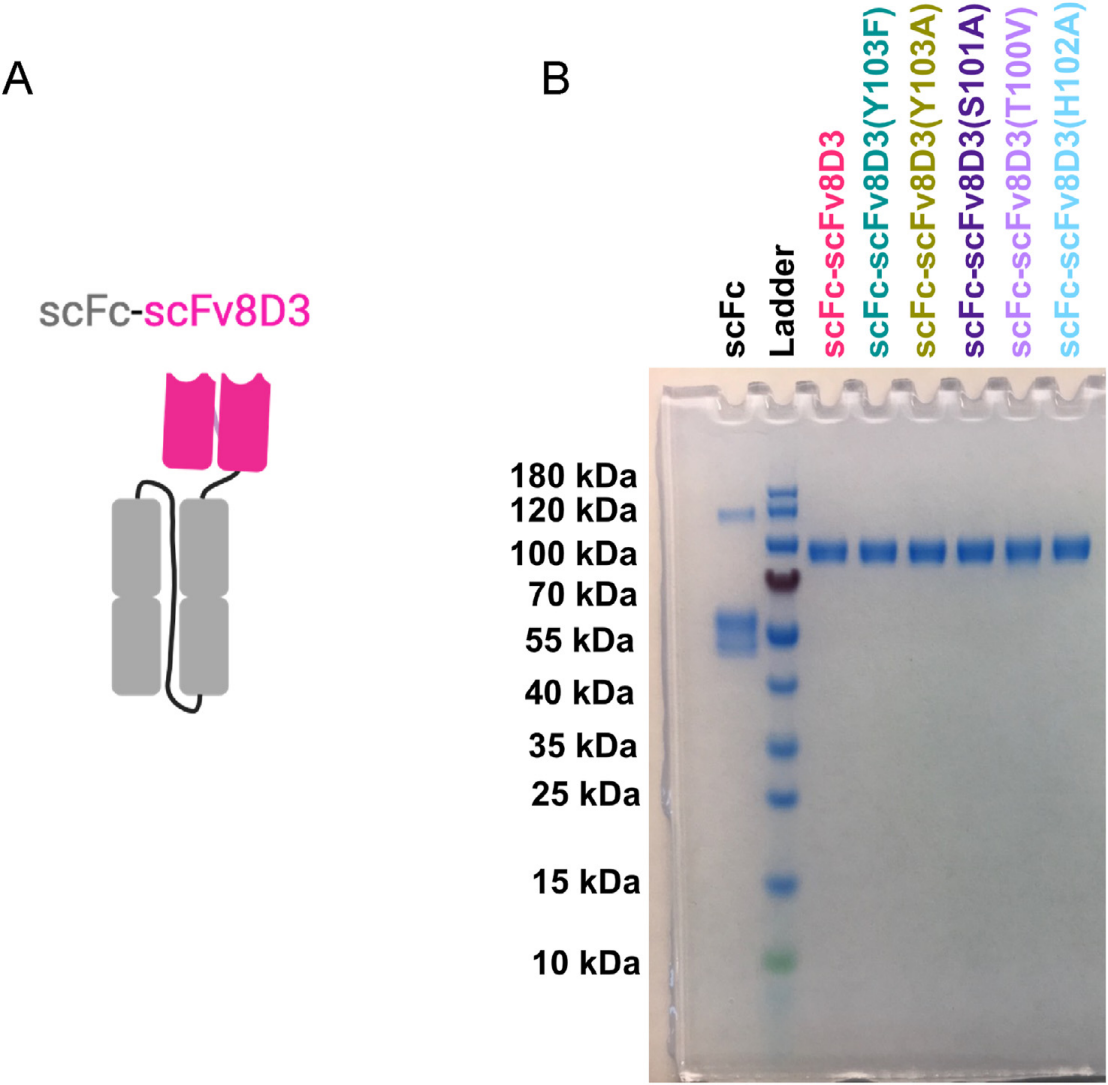
A. Schematic figure of the scFc-scFv8D3 used in the study. A linker was inserted between the two CH1–CH2 domains to create a single chain of the Fc domain, which was then connected to the scFv8D3 via an additional linker. B. Characterization of purity. SDS-PAGE gel analysis of the purified antibody constructs performed in non-reducing conditions, with a pre-stained ladder used to determine the approximate molecular weights of the constructs. Estimation of purity performed with ImageJ is shown in Supplementary Fig. 2.

The scFc-scFv8D3, scFc, and the five scFc-scFv8D3 affinity mutant constructs were expressed in Expi293 ​cells with yields of approximately 1–2 ​mg per liter of transfected culture. SDS-PAGE analysis showed one strong band close to the expected size of 84 ​kDa for scFc-scFv8D3, and the affinity mutant constructs (Fig. 4), with their purity estimated to be close to 100 ​% as no other peaks were visible when analyzing in ImageJ (example shown in Supplementary Fig. 2). The scFc construct showed a strong band at 55 ​kDa representing the monomeric form of the construct, which showed up as a doublet when the sample was applied without boiling or reducing agent. A weaker band below 120 ​kDa represents residual dimer formation following recombinant production of the construct. The purity of the monomeric scFc was estimated to be 86 ​%, with the dimer peak accounting for 14 ​%. During protein-G column purification of constructs containing the scFc, the elution resulted in two peaks, where the first peak represents the monomeric form of the antibody,and the second peak represents multimers formed during the production (example shown in Supplementary Fig. 3). All subsequent experiments were performed with the monomeric form of the antibodies, with the exception of scFc alone, with the purified antibody also containing a proportion of dimers from the second peak (14 ​%). To confirm that the purified scFc-scFv8D3 and scFc-scFv8D3 affinity mutant constructs were monomeric, mass photometry analyses were performed, showing no dimer or multimer formations (Supplementary Fig. 4).

### Decreased mTfR binding shown for all scFc-scFv8D3 affinity mutant constructs

**Fig. 5:**
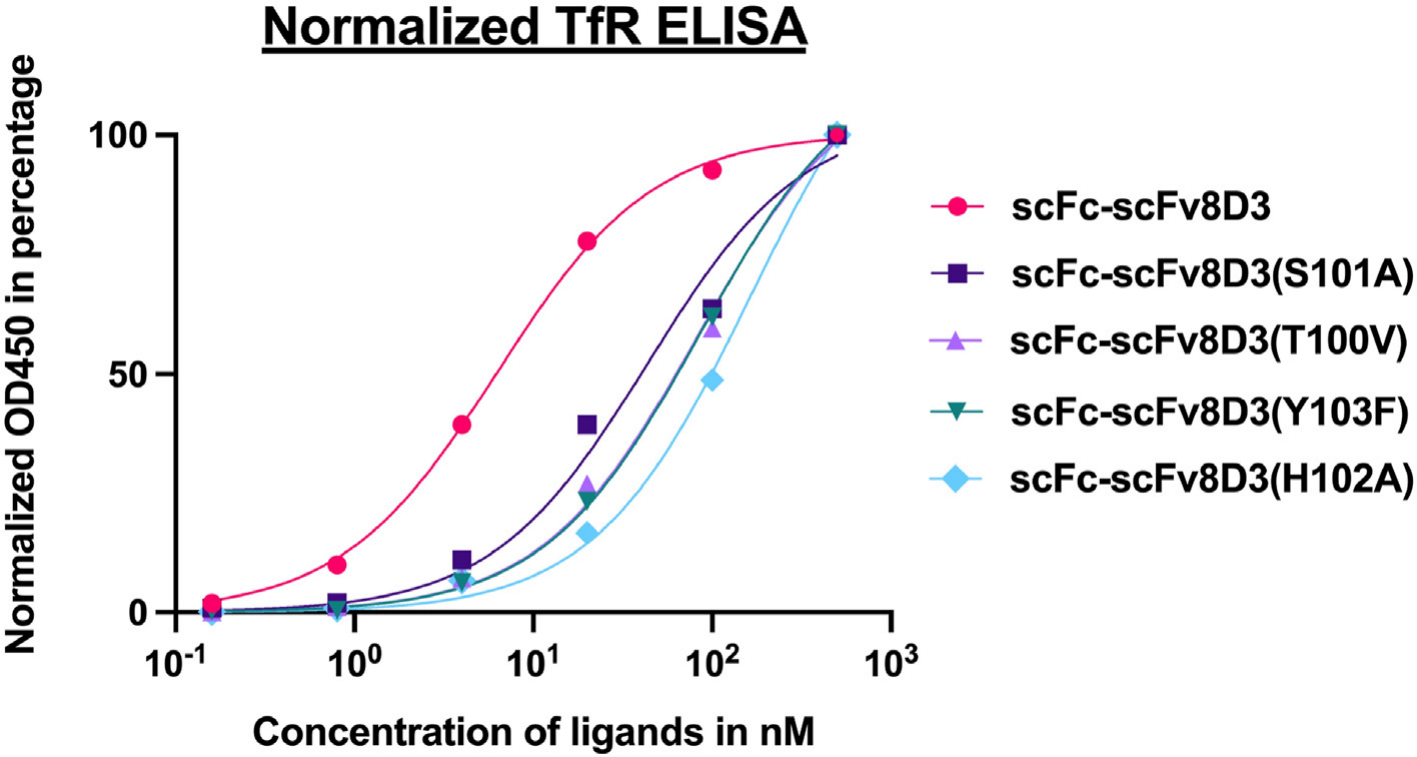
TfR ELISA showing binding efficacy of scFc-scFv8D3 and affinity mutant constructs. Standard curves of each ligand were analyzed in duplicate and detected with an anti-IgG HRP-conjugated antibody recognizing Fc-region, the average response from the duplicates of each antibody concentration are shown in the graph. The binding curves were normalized to maximum binding signal of each construct. Non-linear regression curves were created using a “one site – specific binding” model.

To assess whether the TfR binding strength of the scFc-scFv8D3 affinity mutant constructs was decreased compared to the scFc-scFv8D3 construct, a mTfR-binding ELISA was performed. The majority of the mutant constructs showed decreased binding, while retaining their ability to bind TfR, as can be seen by the positive correlation between increasing ligand concentration and binding of the TfR coated on the ELISA-plate (Fig. 5). The scFc-scFv8D3(Y103A) construct was the only exception, displaying binding only at the highest concentration of 500 ​nM, indicating a non-specific binding pattern (Supplementary Fig. 6). The scFc (negative control), did not show any binding to TfR at all, which was expected since it lacks a TfR binding paratope (Supplementary Fig. 6). The binding curves shown in Fig. 5 are normalized to each respective maximum binding signal to account for differences in max response. For each concentration of all of the ligands two technical replicates were added to the 96-well plate and the averaged response for each concentration are shown in Fig. 5.

### In vitro BBB transcytosis

To study the ability of scFc-scFv8D3 affinity mutant constructs to undergo transcytosis, an in-house developed assay (In-Cell BBB-Trans assay) was used [40]. The In-Cell BBB-Trans assay can robustly demonstrate how well an antibody construct crosses the BBB through transcytosis, while mitigating questions surrounding the half-life effect of the antibody in the bloodstream [40,57]. Due to the inevitable leakage issues related to most, if not all, published in vitro BBB assays, our assay includes a rigorous washing procedure that removes unbound and leaked antibodies following the pulse phase of the assay (Supplementary Fig. 7). Any antibody construct detected in the basolateral chase samples results from antibodies that remain bound to the TfR on the cell layer or endocytosed by the cell during the pulse phase of the assay and subsequentially transcytosed through the cell during the chase phase of the assay.

**Fig. 6:**
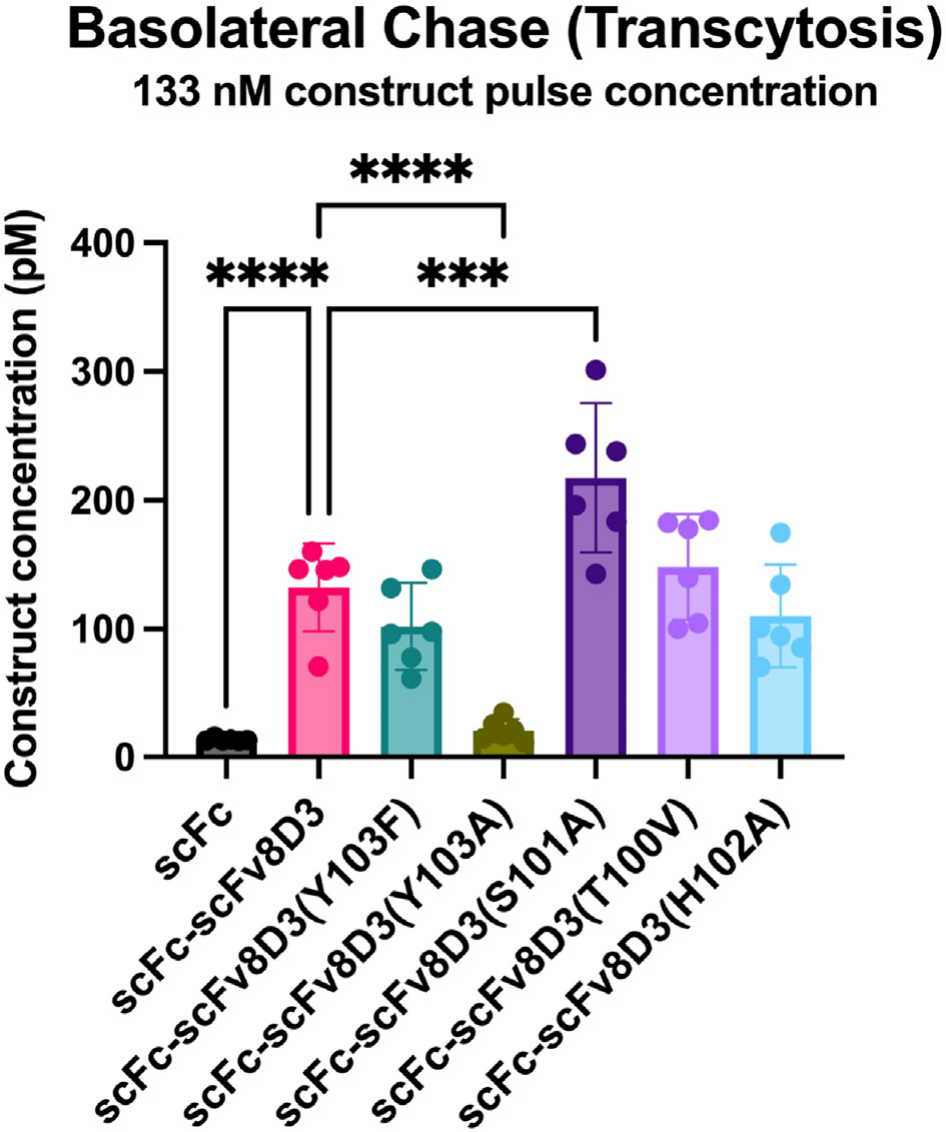
In-Cell BBB-Trans assay using the scFc-scFv8D3 affinity mutants. Graphical representation of average antibody concentrations found in the basolateral 6-h chase compartments of murine capillary endothelial cells plated on 24-well transwell cultures, following a 1-h “pulse” with 133 ​nM scFc, scFc-scFv8D3, scFc-scFv8D3(Y103F), scFc-scFv8D3(Y103A), scFc-scFv8D3(S101A), scFc-scFv8D3(T100V), and scFc-scFv8D3(H102A). Six transwells were used for each construct. The error bars represent 95 ​% confidence intervals. Statistical pairwise comparisons were conducted between scFc-scFv8D3 and the scFc and scFc-scFv8D3 affinity mutant constructs. ∗∗∗ represents a significance level of P ​< ​0.001. ∗∗∗∗ represents a significance level of P ​< ​0.0001.

Using the In-Cell BBB-Trans assay and corroborating in vivo methodologies, we have shown previously that BBB transcytosis is greater for monovalent binding scFv8D3-constructs compared to partially bivalent scFv8D3 constructs when administering at elevated doses (therapeutic dose). In contrast, the opposite is true when administering a lower dose (tracer dose) [57]. Therefore, to test if the affinity mutant constructs were likely to cross the BBB at a therapeutic dose in vivo, we performed an In-Cell BBB-Trans assay using a 133 ​nM pulse concentration of each construct to mimic administering a therapeutic dose in vivo. Using a 6-h chase format, the scFc-scFv8D3(T100A) and scFc-scFv8D3(H102A) constructs showed similar in vitro transcytosis levels to scFc-scFv8D3, while the scFc-scFv8D3(S101A) construct had significantly increased transcytosis (Fig. 6). The scFc-scFv8D3(Y103A) construct was unable to cross the In-Cell BBB- Trans assay, which was expected due to its nearly abolished TfR binding strength (Fig. 5), whereas the Y103F showed almost similar transcytosis compared to scFc-scFv8D3 (Fig. 6). The scFc negative control showed no signs of crossing the cellcular barrier using the assay. The four constructs that demonstrated an ability to undero transcytosis using the In-Cell BBB-Trans assay were selected for further in vivo studies. Due to its inability to undergo transcytosis in vitro, the scFc-scFv8D3(Y103A) was not used in any further studies.

### The half-life of the scFc-scFv8D3 affinity mutants is prolonged in the blood

**Fig. 7:**
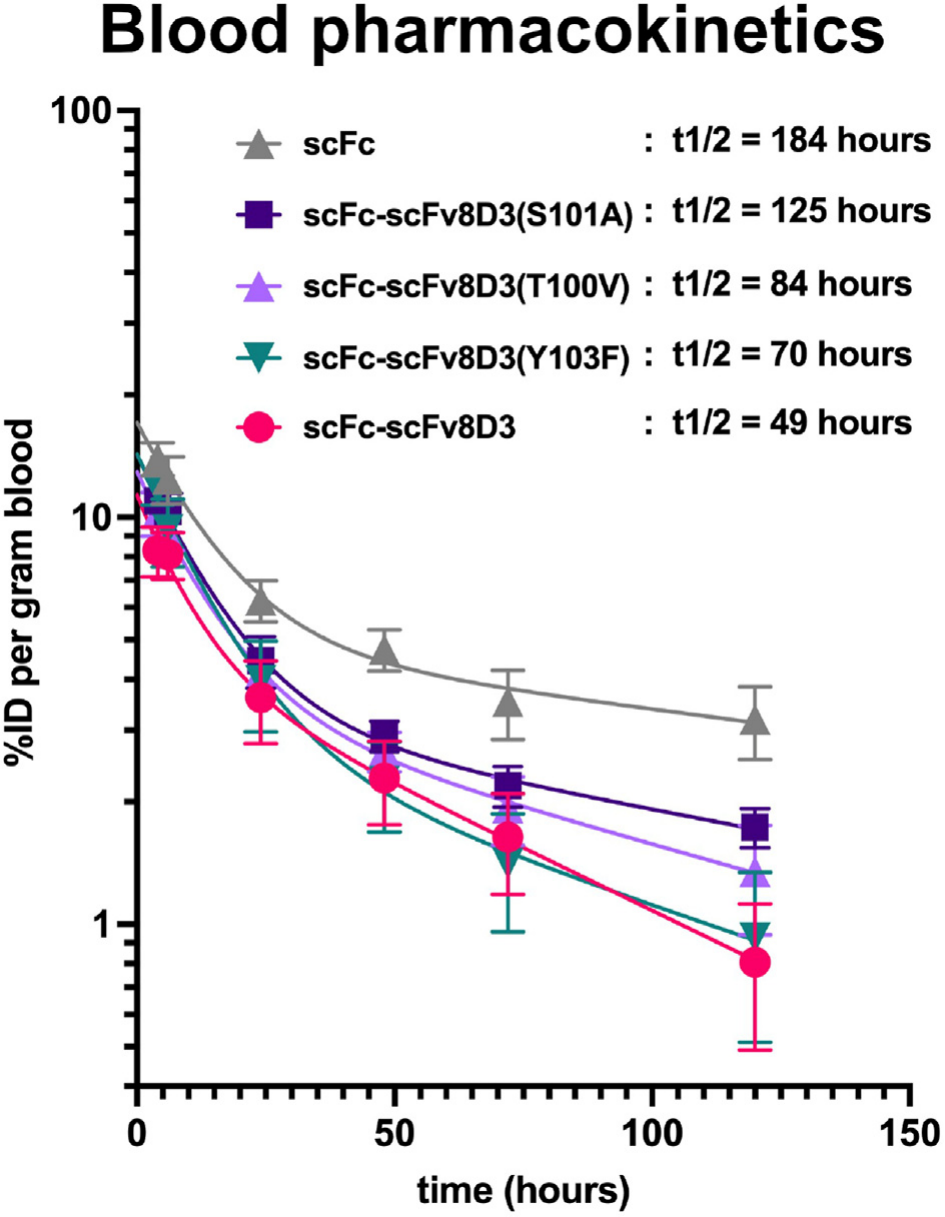
*In vivo* blood pharmacokinetics of ^125^I-labeled scFc-scFv8D3 and affinity mutant constructs in WT mice (n ​= ​4). Blood concentrations expressed as a percentage of injected dose (%ID) per gram blood from blood samples taken at the indicated times, following intravenous injection of the ^125^I radiolabeled constructs at a therapeutic dose (30 ​nmol/kg). 30 ​nmol/kg corresponds to 2.5 ​mg/kg of scFc-scFv8D3 and the scFc-scFv8D3 affinity mutant constructs, while the same nanomolar concentration corresponds to 1.66 ​mg/kg for scFc.

To evaluate if the lowered TfR binding strength of the scFc-scFv8D3 affinity mutants prolonged their half-life in blood compared to scFc-scFv8D3, the constructs were iodine-125 (^125^I)-labeled and intravenously injected at a therapeutic dose of 30 ​nmol/kg bodyweight in wild-type (WT) mice. 30 ​nmol/kg corresponds to 2.5 ​mg/kg of scFc-scFv8D3 and the scFc-scFv8D3 affinity mutant constructs and would correspond to 4.5 ​mg/kg of a normal IgG, while the same nanomolar concentration corresponds to 1.66 ​mg/kg for scFc. This is similar to the concentration used for therapeutic experiments using this BBB transporter [25,58]. The scFc-scFv8D3(H102A) affinity mutant displayed a propensity to partly multimerize when concentrated to 7.5 ​μM in order to reach a sufficiently high concentration to perform animal experiments (Supplementary Fig. 5). The multimerization of the concentrated scFc-scFv8D3(H102A) was reflected in higher TfR binding strength indicative of binding avidity (Supplementary Fig. 8). Due to this observation, the in vivo and ex vivo results of scFc-scFv8D3(H102A) affinity mutant are shown only in the supplementary materials. Blood samples were taken at the indicated time points over a five-day period and gamma radiation levels were measured to determine the blood concentration each construct over time (Fig. 7). The calculated half-lives of all of the scFc-scFv8D3 affinity mutants were prolonged compared to scFc-scFv8D3 half-life, which was calculated to be 49 ​h. The half-life of scFc-scFv8D3(S101A), scFc-scFv8D3(T100V), scFc-scFv8D3(Y103F) and scFc-scFv8D3(H102A), was calculated to be 125, 84, 70 and 64 ​h respectively (Fig. 7 and Supplementary Fig. 9). Performing a Tukey's multiple comparison test revealed a significantly longer half-life for scFc-scFv8D3(S101A) when compared to scFc-scFv8D3 (p ​= ​0.004). The half-life of scFc was calculated to be 184 ​h (Fig. 7).

At the end of the 120-h blood-half life in vivo experiment, the gamma radiation levels in the brains of the mice were measured ex vivo to evaluate if the scFc-scFv8D3 affinity mutants also had increased brain concentration compared to scFc-scFv8D3 at this time point (Supplementary Fig. 10). The signal measured at this time point was bordering the detection limit of the gamma counter, indicating that very little of the scFc and scFv8D3-constructs remained in the brain at this later time point. This was unexpected as the half-life of the mutants showed that their concentration in blood was still high. This indicates that half-life and affinity of antibodies are not the only factors that determine the efficacy of brain-uptake. The biodistribution at the end of this experiment is shown in Supplementary Fig. 12.

### scFc-scFv8D3 affinity mutants exhibit significantly higher brain concentration after 24 ​h

**Fig. 8:**
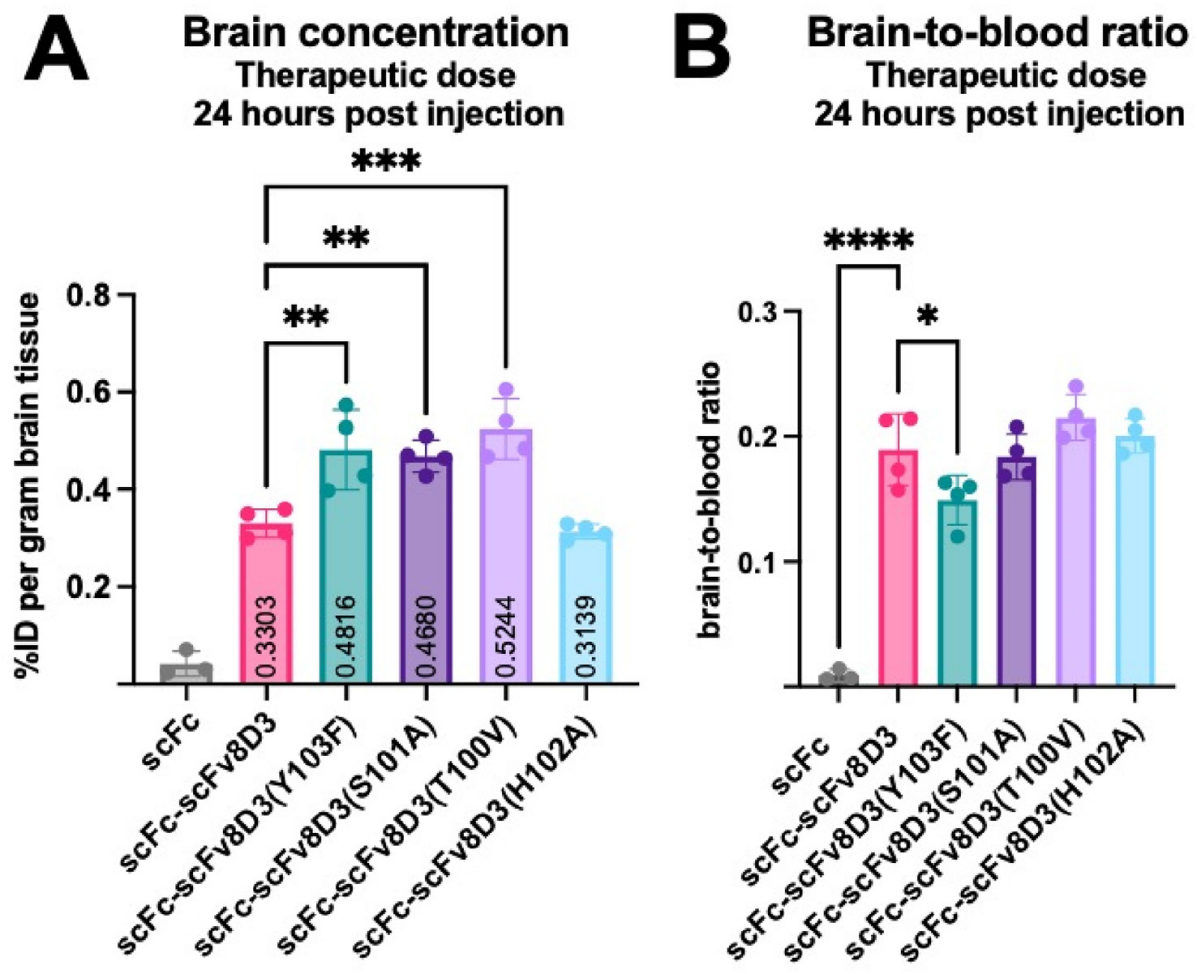
A. Brain concentration and B. Brain-to-blood ratios of ^125^I-labeled scFc-scFv8D3 and affinity mutant constructs in WT mice 24 ​h post injection. Brain uptake concentrations expressed as a percentage of injected dose (%ID) per gram of brain tissue from brains collected and measured for radioactivity ex vivo 24 ​h post-injection, following intravenous injection of the ^125^I radiolabeled constructs at a therapeutic dose (30 ​nmol/kg). 30 ​nmol/kg corresponds to 2.5 ​mg/kg of scFc-scFv8D3 and the scFc-scFv8D3 affinity mutant constructs, while the same nanomolar concentration corresponds to 1.66 ​mg/kg for scFc. Results are presented as mean ​± ​SD. Statistical pairwise comparisons were conducted between scFc-scFv8D3 and the scFc and scFc-scFv8D3 affinity mutant constructs. ∗ represents a significance P ​< ​0.05, ∗∗ represents P ​< ​0.01, and ∗∗∗ represents P ​< ​0.001.

To overcome our inability to detect antibodies *ex vivo* following an extended 120-h blood half-life study, a shorter 24-h *ex vivo* experiment was performed to assess brain uptake of the affinity mutant constructs following intravenous administration at therapeutic doses. At the end of the 24-h experiment, the gamma radiation levels in the brains of the mice were measured. The scFv8D3(Y103F), scFc-scFv8D3(S101A), and scFc-scFv8D3(T100V) affinity mutants had significantly higher brain concentration compared to scFc-scFv8D3 (Fig. 8), with similar brain concentrations observed for all three mutant constructs. The fourth, likely partly multimerized, affinity mutant scFc-scFv8D3(H102A) had similar brain concentration to that of scFc-scFv8D3 (Supplementary Fig. 11). The negative control scFc had a very low brain concentration of 0.04 %ID/g, which was expected as it lacks the BBB shuttle scFv8D3 (Fig. 8). The biodistribution at the end of this experiment is shown in Supplementary Fig. 13.

## Discussion

As of yet, the most promising strategy for delivering antibody therapeutics into the brain is to target TfR [[8], [9], [10]], where one of the most widely studied TfR-binders is the high affinity anti-mouse TfR antibody 8D3 [13,16,22,[25], [26], [27], [28], [29], [30]]. The binding strength to TfR can affects both the amount of crosslinking on the BBB, release in brain parenchyma and half life in blood. To be able to start to delineate the impact of the affinity and the increased exposure due to extended half life a combination of *in vitro* and *in vivo* studies is needed. In the *in vitro* system blood half-life has no influence and show on transcytosis, while in vivo blood half-life has an effect. In the present study, we have utilised an in-house designed monovalent binding scFv8D3 antibody connected to murine scFc from IgG2c [57]. Mutations were then made to the scFv8D3 in order to generate constructs with different binding affinities. The TfR binding properties of 8D3 has been extensively used for the purpose of brain delivery and it has been used in varied antibody formats with different TfR binding valencies, such as monovalent [26,59], bivalent [16,[28], [29], [30]]^,^ and partly bivalent [13,22].

Interestingly, even though previous studies have focused on deducing the connection between reducing affinity and transport into the brain using other monovalent TfR binding antibody formats, this study is the first of its kind in which the effect of decreasing 8D3s TfR affinity has been studied using a monovalent 8D3 antibody format. The In-Cell BBB-Trans assay showed significantly greater transcytosis for scFc-scFv8D3(S101A) compared to both scFc-scFv8D3 and the other affinity mutants, indicating that the endocytosis and/or the intracellular sorting toward transcytosis of the mutant is enhanced.

These results indicate that reducing TfR binding affinity could join increased blood half-life and reduced cross-linking of the TfR as a dependent factor that controls brain uptake of therapeutic antibodies. It has been shown previously that decreasing the affinity of high affinity anti-TfR antibodies improves brain delivery of both bivalently and monovalently binding TfR-antibodies [16,21,31,60]. Furthermore, as monovalent binders cannot bind with avidity, they have faster dissociation rates than the corresponding bivalent binder [34], potentially providing monovalent TfR binders with an additional advantage. Other key processes that could contribute to brain uptake efficacy of monoclonal antibodies, which are not covered in this study, are intracellular sorting, dissociation at suitable times and lysosomal escape. Relevant to our findings, Do et al. reported a 7-fold increased dissociation rate for their bivalent affinity mutant with the same single mutation [31]. Therefore, it is possible that our scFc-scFv8D3(S101A) construct also has an increased dissociation rate, which in turn could enhance release and lead to improved brain uptake. No linear correlation between reducing TfR affinity and increasing half-life was observed for the affinity mutants used in the study. Similar results have been reported by Webster et al. and Do et al. where their bivalent 8D3 affinity mutants generally exhibited prolonged half-life, but no linear correlation was observed [16,37].

When we investigated the brain delivery of our scFv8D3 affinity mutants, we observed that all of the affinity mutants, except the likely multimerized scFc-scFv8D3(H102A), had increased brain uptake 24-h post-intravenous injection compared to the wildtype scFc-scFv8D3 construct. However, results using the In-Cell BBB-Trans assay revealed only scFc-scFv8D3(S101A) as having enhanced transcytosis activity compared to the wildtype construct. After 24-h, all three mutants showed enhanced brain delivery compared to the control. One explanation to these contrasting results revolves around measurement of the radioactivity in the brain cannot distinguish between actual parenchymal location of the antibody compared to antibody still bound at the BBB. It could be that the scFc-scFv8D3(S101A) has a more efficient transcytosis, but is bound less to the BBB due to a lower affinity to the TfR, resulting in the detected signal more likely localized to the brain parenchyma. Conversely, the other constructs have a higher affinity to the BBB, which is responsible for the equally elevated radioactivity levels in the brain samples. If this assumption is true, one could speculate that the actual increase in transcytosis of scFc-scFv8D3(S101A) is even higher than that measured in vivo since the scFc-scFv8D3 is the construct with the highest affinity and hence likely to be bound the most to the BBB. In the In-Cell BBB-Trans assay it is only the amount of antibodies that are bound to or inside the cells at the end of the chase that is measured, the ones that were transported across the cells before this time point are washed away. This can also be a reason for slightly different results in the two assays. Some construct might be more efficient in crossing the BBB initially but with a decreased rate later on (due to changed expression levels of TfR for instance).

Very little of our constructs were left in the brain 120-h post-intravenous injection, including the scFc-scFv8D3(S101A) mutant. At this time point none of the mutants had increased brain concentration compared to the negative control, scFc. The fact that the brain concentration observed for scFc without the scFv8D3 is similar to that at 24 ​h post-injection may be due to the fact that it is not actively transported in or out of the brain and lacks a target in the blood, while also having a long blood half-life. We have also seen indications that the antibodies might still be in the brain but that the iodine has been removed and cleared from both the cells and the brain. In experiments where we use radiometals such as 111 Indium instead we get much higher signals [61]. These stays in the cells after degradation, and hence give a value of how much that has been in the brain since the injection and not at a given timepoint only. It is also possible that most of the uptake into the brain happens initially and that the rate later is much slower.

Our 24-h *in vivo* data supports the hypothesis that the strategy of fine-tuning mTfR affinity of monovalently binding 8D3-constructs is advantageous, similar to applying the strategy to bivalent 8D3 constructs. As of yet, the most convincing data supporting the effectiveness of the strategy for bivalent 8D3 constructs was reported by Webster et al., where the authors generated affinity mutants with 56-, 130-, and 610- nM affinity [16], which they named 8D3_56,_ 8D3_130,_ and 8D3_610_ respectively. After injecting WT mice with 20 ​mg/kg of each construct, they measured the brain uptake over a 2-week period, and they found that all three 8D3 affinity mutants had greatly increased brain concentration, where the 8D3_130_ the exhibited the highest brain delivery followed by 8D3_56_ and lastly 8D3_610_. Remarkably, the 8D3_130_ mutant achieved a 13-fold increase in maximum observed concentration (C_max_) and a 70-fold increase in area under the curve (AUC) compared to the original 8D3. The 8D3_56_ mutant had similar C_max_ but about half the AUC compared to the 8D3_130_ mutant, while the 8D3_610_ mutant had a 2-fold decrease in C_max_ and a 3-fold decrease in AUC compared to the 8D3_130_ mutant. These publications corroborate the findings in our study, even though the difference in affinity of our constructs is smaller.

In conclusion, we successfully created affinity mutants of scFv8D3 with lowered TfR affinity, longer blood half-life and exhibiting significantly higher brain concentration 24-h post-injection. These findings were mimicked using an *in vitro* BBB transcytosis model system, with the uptake of scFc-scFv8D3(S101A) showing a significant increase compared to wildtype controls. Our data supports the practice of fine-tuning the affinity of TfR antibodies for both bivalent and monovalent 8D3 constructs alike to improve brain uptake of monoclonal antiboides. As monovalent BBB shuttles are increasingly employed for delivering antibodies to the brain, the findings of our study provides a valuable resource for guiding much needed future endeavors into this field of neurological therapeutic research.

## Materials and Methods

### Amino acid functional group analysis

To identify the AAs within the VHCDR3 region with the highest potential to form strong interactions with the mTfR epitope, the functional groups of the AAs within the respective regions were analyzed based on their theoretically possible interactions. Specifically, the potential to form any of the following strong interactions were looked for; ionic interactions [46], hydrogen bonding, conventional and hydrogen – π, (H– π) [[46], [47], [48]], π – π interactions [46,48,49], cation – π interactions [46,48,50,51], anion – π interactions [46,48,[52], [53], [54]] and sulfur – π interactions [46,48,55,56]. The AAs of 8D3s mTfR epitope (200 QNMVTIVQSNGNLDPVES 217) [23] (Fig. 1B) (numbered from the N-terminal of the whole mTfR protein (Uniprot Database [62] entry Q62351)), and the VHCDR3 region of scFv8D3 (99 PTSHYVVDV 107) [28] (Fig. 2) are numbered from the N-terminal heavy chain of the scFv.

### In silico protein-protein docking analysis

Protein-protein docking analysis was also performed to identify AAs within the VHCDR3 region of scFv8D3 likely to contribute to mTfR affinity. To enable the protein-protein docking, a shortened mTfR peptide, but retaining the secondary structure of scFv8D3s epitope, ranging from Q200 to K380, was modeled using AlphaFold v2 colab [45,63]. Additionally, a homology model of scFv8D3 was built using SWISS-MODEL [64]. Standard settings were used for both AlphaFold and SWISS-MODEL to generate the models. Then, scFv8D3 was docked with the mTfR peptide in ezCADD:s ezPPDock [65] and AbAdapt [66]. In ezPPDock, all AA except the epitope (P218 – K380) of the shortened mTfR peptide were blocked from interaction, and all AAs except the CDR regions ​± ​2 AAs upstream and downstream of the scFv8D3 model were similarly blocked. The XTC setting was selected as the trajectory format for the docking. Out of the 2001 generated poses, 15 were selected by visual inspection in PyMOL [67], with the inclusion criteria of having at least one AA of the scFv8D3 VHCDR3 within interaction distance of the mTfR epitope. Interaction distance was defined as being within 4.5 ​Å. In AbAdapt, the VH and VL chains of scFv8D3 were inputted separately as AbAdapt only supports that format, and subsequently, the docking was performed with standard settings. 30 of the first 31 poses generated by AbAdapt were selected based on having the recommended score of ≥20 where scoring reflects Piper-Hex co-clustering [66].

### Creation of scFc-scFv8D3 affinity mutants

The affinity mutants were based on our previously described single-chain monovalent BBB transporter, consisting of a single-chain Fc antibody (scFc) conjugated to scFv8D3 (scFc-scFv8D3) [57]. To create affinity mutants of scFc-scFv8D3, single point mutations were introduced in the VHCDR3 of scFv8D3, with specific AAs targeted based on the results of the AA functional group analysis and the *in silico* protein-protein docking analysis. All of the genes for the constructs used in this study were synthesized and cloned into the pcDNA3.4 vector by Thermofisher (GeneArt, Regensburg, Germany).

### Expression and purification of the antibody constructs

The antibody constructs used in the experiments were expressed as described in earlier published work [13,68] using Expi293 ​cells (Thermofisher cat. no. A14527) transiently transfected with pcDNA3.4 vectors using polyethylenimine (PEI – Polyscience cat. no. 24765–1) as the transfection reagent. The antibody constructs were purified on a protein G column (Cytiva cat. no. 17-0405-01), and to separate monomeric protein from dimeric and multimeric protein, the elution was done with a shallow 40 column volumes gradient of 0.7 ​% acetic acid. The gradient elution resulted in a first peak containing the monomeric protein and a second separate peak containing dimeric and multimeric protein. Fractions from the first peak were then carefully concentrated with Amicon centrifugal filters (Sigma-Aldrich cat. no. UFC501024), and buffer exchanged to PBS with Zeba spin columns (ThermoFisher cat. no. A44301). The concentrations of the purified antibody constructs were determined by measuring their absorbance at 280 ​nm (nm) with a spectrophotometer instrument (Denovix, DS-11 Series, USA) and calculating their concentrations by factoring in their molecular weight and molecular extinction coefficients.

### Confirmation of purity and size of the antibody constructs

To confirm the size and purity, the purified antibody constructs were analyzed using SDS-PAGE followed by PAGE blue protein staining. Briefly, the antibody constructs were mixed with LDS sample buffer (Life Technologies cat. no. B0007) and loaded, without adding reducing agents and without boiling, on Tris 4 ​%–12 ​% 15-well precasted gels (Invitrogen cat. no. NW04125BOX). The gels were run for 1.5 ​h in MES running buffer (Thermofisher cat. no. NP0002) at 80 ​V and then stained with PAGE blue protein staining solution (Thermo Fisher Scientific cat. no. 24620). After staining, the gels were rinsed with deionized water and images of the gels were taken with an Odyssey Fc instrument (LI-COR Biosciences) with Image Studio software (version 5.2.5). A 10–180 ​kDa PageRuler™ Prestained Protein Ladder (Thermofisher cat. no. 26616) was used as a molecular weight standard to confirm the correct size, and the gel images were analyzed with Fiji (ImageJ) to determine the purity of the constructs.

### Mass photometry

Mass photometry analyses were performed on a Refeyn 2 ​MP mass photometer (Refeyn Ltd) calibrated with NativeMark Unstained Protein Standard (Thermo Fisher Scientific cat. no. LC0725). The proteins were mixed with PBS, giving final concentrations between 7.5 ​nM and 46.5 ​nM prior to analysis.

### Assessing in vitro binding of antibody constructs to mouse transferrin receptor

The binding of the antibody constructs to mTfR was assessed by a previously described indirect mTfR ELISA [69]. In brief, 96-well half area plates (Corning Incorporated cat. no. 3960) were coated with 50 ​ng/well with recombinant mouse TfR extracellular domain protein (prepared in our lab) in PBS (Thermofisher cat. no. 18912014), and stored overnight at 4 ​°C. The plates were then blocked for 2 ​h at room temperature (RT) with 1 ​% BSA in PBS while shaking at 500 ​rpm. After blocking, serial dilutions of the antibody constructs were added in duplicates and for each concentration of the antibody constructs the averaged response of the two technical replicates were used. The samples were incubated for 2 ​h at RT while shaking. For detection, a goat anti-mouse antibody conjugated to horse-radish peroxidase (HRP) (Sigma cat. no.12349) was used. The signal development was done with K-blue aqueous TMB (Neogen Corp cat. no. 331177), and the absorbance was measured at 450 ​nm using a microplate reader (Spark® multimode microplate reader, Tecan). The dilution series of antibody constructs were made in ELISA incubation buffer (1x PBS with 0.1 ​% BSA and 0.05 ​% Tween-20 (Sigma cat. no. P9416)), and the wells were washed with ELISA washing buffer (1 ​× ​PBS with 0.05 ​% Tween-20) in-between each step.

### Determination of in vitro blood-brain barrier transcytosis of antibody constructs

To screen the antibody constructs before performing in vivo brain uptake experiments, the previously described In-Cell BBB-Trans assay [40] was performed to assess the *in vitro* transcytosis efficacy of the antibody constructs. In short, the pulse-chase experiments were performed with Bio-One Thincert™ translucent (1 ​× ​10^8^ pores/cm2, Greiner cat. no. 662640) PET membranes (transwell) with high-density 0.4 ​μm pores. The transwells were coated with 90 ​000 murine cerebral endothelial cells (cEND) (Applied Biological Materials cat. no. T0290) in 24-well cell culture plates (BioNordika cat. no. 662640), and incubated for 4 ​h at 37 ​°C and 5 ​% CO_2_ in complete DMEM medium (Gibco™ cat. no. 11960044) with various supplementations all of which from Gibco™: 10 ​% FBS (cat. no. 10270106), 1 ​× ​Glutamax (cat. no. 35050061), 1 ​mM of sodium pyruvate (cat. no. 11360039), 1 ​× ​non-essential amino acids (cat. no. 11140–050), and 10 ​U/ml of penicillin/streptomycin (cat. no. 15140122). After 4 ​h, the cells had their incubation medium exchanged for serum-free medium (DMEM supplemented with 1x non-essential amino acids, 1x Glutamax, 1 ​mM of sodium pyruvate, and 10 ​U/ml of penicillin/streptomycin) and were incubated for ∼72 ​h before the start of the experiment. The pulse-phase of the experiment was started by exchanging each transwell to fresh serum-free medium. Serum-free medium containing 133 ​nM of each antibody construct was added to the apical compartment of each transwell. The pulse-incubation was done for 1 ​h at 37 ​°C and 5 ​% CO_2_, with six technical replicates for each condition. After the pulse, the media from the apical and basolateral compartments was collected and the cell monolayers were washed three times apically and basolaterally at RT with serum-free media. The media used for the third wash was also collected (wash samples). After collecting the wash samples, the chase-phase was started by the addition of fresh serum-free medium to the apical and basolateral compartments and incubating for 6 ​h at 37 ​°C and 5 ​% CO_2_, after which the medium was collected from both compartments (chase samples).

### Analysis of media samples from the In-Cell BBB-Trans assay

To analyze the transcytosis efficiency of the antibody constructs, the collected samples from the In-Cell BBB-Trans assay were analyzed by a sandwich ELISA as previously described [40]. In brief, 96-well ELISA plates were coated overnight at 4 ​°C with 1:5000 (v/v) with a Goat-anti Mouse IgG, F(ab’)2 fragment specific capture antibody (JacksonImmunoResearch, cat. no. 109-005-097) diluted in PBS. The plates were then blocked for 2 ​h at RT with 1 ​% BSA in PBS while shaking at 500 ​rpm. After the blocking, samples from the In-Cell BBB-Trans assay were incubated together with known standard concentrations of the antibody constructs for 2 ​h at RT while shaking at 500 ​rpm. For detection, signal development, and signal measurement, the same materials and procedures as for the indirect mTfR ELISA described above were used. The wells were washed with ELISA washing buffer (1x PBS with 0.05 ​% Tween-20) between each step.

### Radiochemistry

For the in vivo experiment, equimolar amounts of the antibody constructs were labeled with iodine-125 (^125^I) using Chloramine-T as described previously [57,70]. Briefly, the antibody constructs were mixed with ^125^I (Perkin Elmer Inc) stock solution directly ionized with Chloramine-T (Sigma Aldrich cat no. 857319) in PBS and incubated for 90 ​s. The reaction was then stopped with 1 ​mg/mL sodium meta-bisulfite (Sigma Aldrich, cat. no. 08982). The labeled antibody constructs were purified from unbound free ^125^I using Zeba columns (VWR cat. no. 17-0853-02) and eluted in PBS for buffer exchange. The radiolabeling was performed within 2 ​h of starting the *in vivo* experiment. The radiolabeling yield, calculated based on the amount of initially added ^125^I and on the remaining activity of the radiolabeled antibody constructs after buffer exchange, was between 65 -75 ​%. To limit the animal's exposure to radioactivity, only 10 ​% of the administered therapeutic dose (30 ​nmol/kg) was ^125^I-labeled by mixing unlabeled antibody constructs at a 1:10 (v/v) ratio.

### Animals

For the animal experiment, 3.5 months old wild-type male mice were used (C57BL/6JBomTac, purchased from the certified supplier Taconic M&B). Animals were housed in an animal facility at Uppsala University in rooms with controlled temperature (20–22 ​°C) and humidity (50–55 ​%), with individually ventilated cages (2–4 animals/cage). The animals had free access to food and water and had daily surveillance by trained personnel. The procedures described were performed according to the Swedish ethical policies regarding animal experiments and approved by the Uppsala County Animal Ethics Board (#5.8.18-04903-2022). All efforts were made to reduce the number of animals used and to minimize the animal's exposure to stress and suffering.

### Blood pharmacokinetics and biodistribution in wild-type mice

Blood pharmacokinetsics and biodistribution was investigated in 3.5 months old C57Bl/6JBomTac WT mice (n ​= ​4). An intravenous injection (tail vein) of a therapeutic dose (30 ​nmol/kg) of each ^125^I-labeled antibody construct was performed. No blinding or randomization was used for the experiment, but different experimental groups were distributed equally among the cages. Blood samples (8 ​μl capillaries, Vitrex Medical cat.no. 172613) were obtained from the tail vein at 4-, 6-, 24-, 48-, and 72-h post-injection. Whole blood half-lives were calculated by using Prism 10 software (GraphPad Software, Inc., La Jolla, CA, USA) using a nonlinear regression with a two-phase decay model, where the plateau was constrained to zero. At the conclusion of the experiment, 120-h post-injection, the mice were anesthetized with 3 ​% isoflurane and euthanized by transcardial perfusion with 0.9 ​% saline. Terminal blood was collected from the heart prior to transcardial perfusion and centrifugated at 15.000×*g* for 5 ​min to separate plasma from blood cells. Brains, peripheral organs (liver, spleen, heart, lung, kidney, pancreas), and tissues (bone, skull) were isolated after perfusion, and their radioactivity levels were measured using a gamma counter (WIZARD 1480, Wallac Oy, Turku, Finland) as previously described [25]. The concentration of the antibody constructs was then quantified based on the measured radioactivity as a percentage of injected dose (%ID) per gram of blood, organ, or tissue respectively.

### Biodistribution in wild-type mice 24 ​h post-injection

Biodistribution was investigated in 3.5 months old C57Bl/6JBomTac WT mice (n ​= ​4) for each group except the negative control group (n ​= ​3) by intravenous injection of a therapeutic dose (30 ​nmol/kg) of each ^125^I-labeled antibody construct into the tail vein. No blinding or randomization was used for the experiment. Blood samples (8 ​μl capillaries, Vitrex Medical cat.no. 172613) were obtained from the tail vein at 2-, 6-, and 24 ​h post-injection. Plasma was collected at 6 ​h post-injection. Euthanasia of the animals and dissection of the animals as well as sample collection and radioactivity measurement were all performed in the same manner as described in Blood pharmacokinetics and biodistribution study described above.

### Statistical analysis

For ELISA and blood pharmacokinetics no statistical tests or comparisons were made. For ELISA non-inear regression was performed using the “One site – Specific binding”. For blood pharmacokinetics non-linear regression with “Two phase decay” was performed with “Least squares regression” fitting and Goodness-of-fit: R squared (R squared values were between 0.98 and 0.99). Data in bar graphs are presented as mean ​± ​95 ​% confidence intervals for the In-Cell BBB-Trans assay or mean ​± ​standard deviation (SD) for brain uptake and biodistribution bar graphs. The data was tested for normality (gaussian distribution) by performing a Shapiro–Wilk test using an *α* value of 0.05. All determined values demonstrated normal, and the data was therefore analyzed by One-way ANOVA statistical test, applied for each of the scFc-scFv8D3 affinity mutants compared to scFc-scFv8D3, with Dunnett's multiple comparison correction for p-values: (∗) <0.05, (∗∗) <0.01, (∗∗∗) <0.001, and (∗∗∗∗) <0.0001. The tests were performed using Prism 10 for MacOS version 13.5.

## Authors’ contributions

GH and ADR designed the project. GH, ADR, and JE created the protein constructs. AG transfected all the proteins. ADR and JE purified the protein constructs. ADR, JM, and JE performed the in vitro assays. NM, ADR, and JM performed in vivo work. GH, ADR, NM, JM, and JE analyzed the results. ADR, GH and JM wrote the manuscript with valuable input from all the co-authors. The authors read and approved the final manuscript.

## Data availability statement

The datasets used and/or analyzed during the current study are available to the corresponding author upon reasonable request.

## Declaration of competing interest

The authors declare that they have no known competing financial interests or personal relationships that could have appeared to influence the work reported in this paper.
