# Supplementary material for: Lowering the affinity of single-chain monovalent BBB shuttle scFc-scFv8D3 prolongs its half-life and increases brain concentration
Source: Neurotherapeutics. 2024 Dec 4;22(1):e00492. doi: 10.1016/j.neurot.2024.e00492 (PMC11742849; doi:10.1016/j.neurot.2024.e00492)
Supplement: Multimedia component 1 [file mmc1.docx]

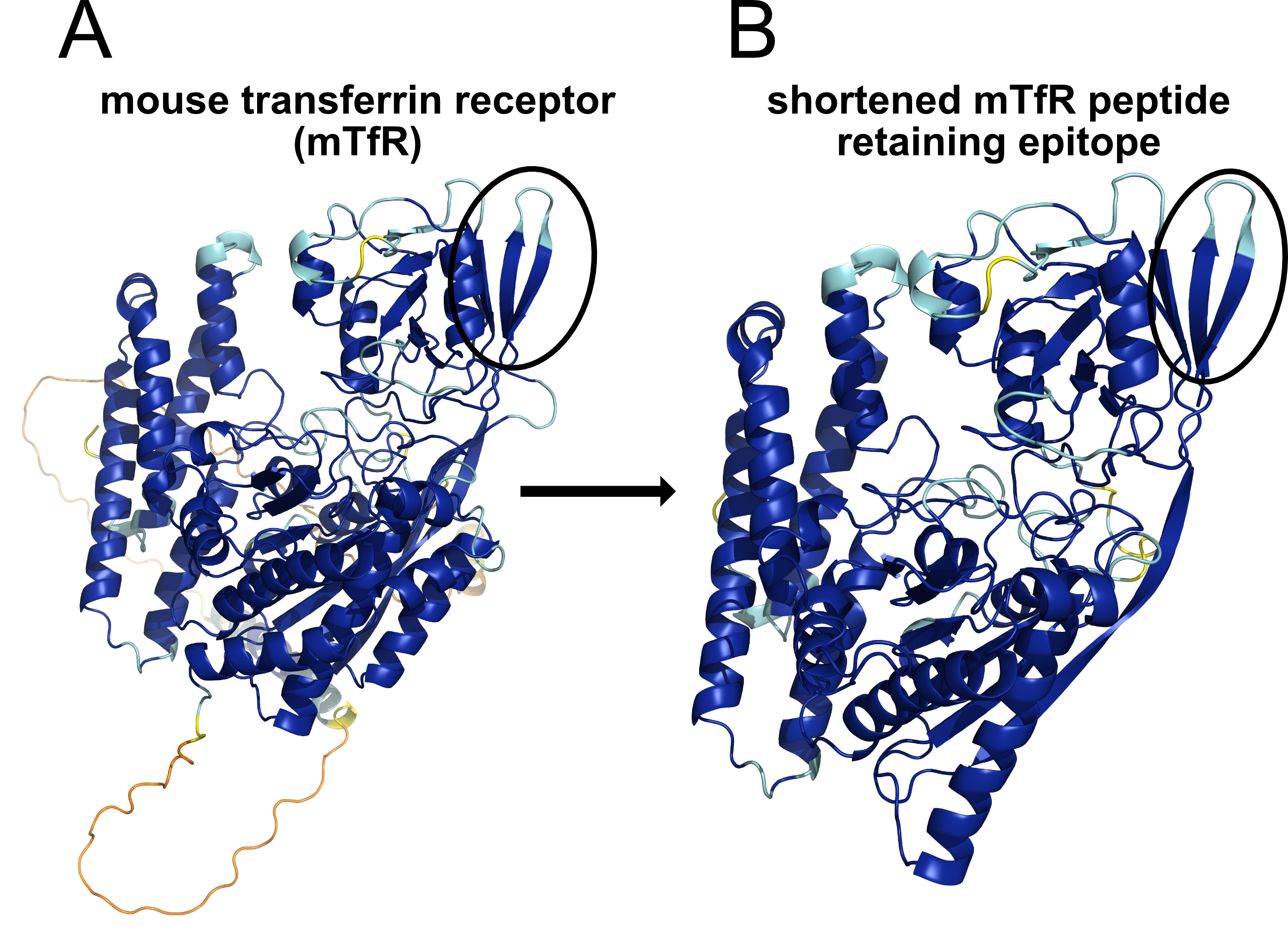


**Supplementary figure 1.** Model of mTfR and shortened mTfR peptide. **(**A). mTfR modeled by AlphaFold2 with scFv8D3 epitope highlighted. (B). shortened mTfR peptide modeled by AlphaFold2 retaining the epitope scFv8D3. A and B are colored according to AlphaFold2s predicted local distance difference test (pLDDT) score; blue = pLDDT > 90, turquoise = 90 > pLDDT > 70, yellow = 70 > pLDDT > 50, orange = pLDDT < 50.

**
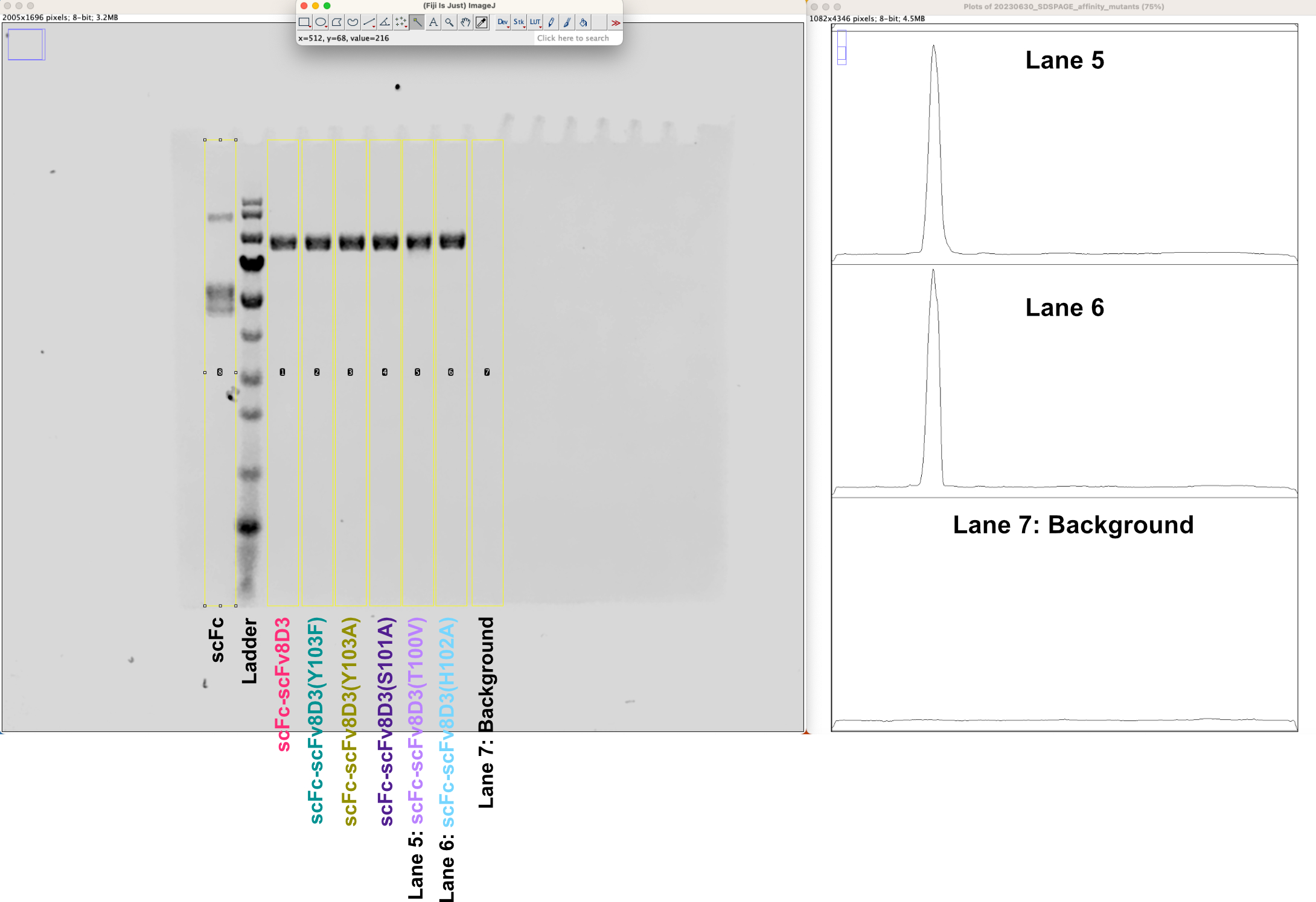
**

**Supplementary figure 2.** Example of purity estimation of scFc-scFv8D3 constructs. The purity of each construct used was estimated by ImageJ (Fiji) with no other peaks visible for any of the scFc-scFv8D3 constructs.


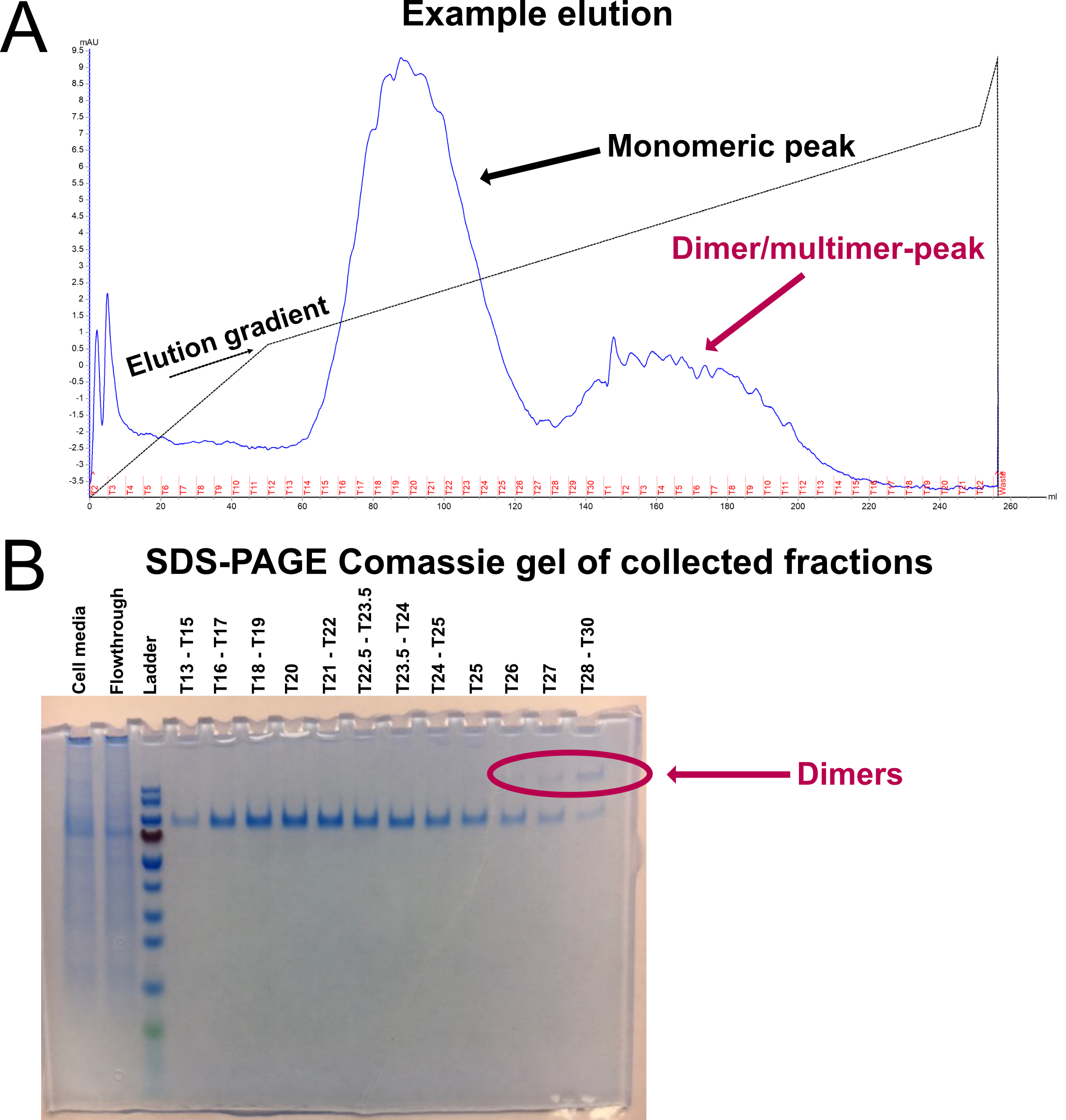


**Supplementary figure 3.** Example of protein-G elution of scFc-scFv8D3 construct, Äkta purification spectrogram and SDS-PAGE gel analysis. (A). Äkta purification spectrogram of example protein-G elution. During protein-G column purification of constructs containing the scFc the elution results in two peaks where the first peak consists of monomers, and the second peak consists of dimers and multimers formed during the production. (B). SDS-PAGE Coomassie gel of collected fraction showing the monomeric peak collected between fractions 13 to 25 and the dimer/multimer-peak collected between fractions 26-30.


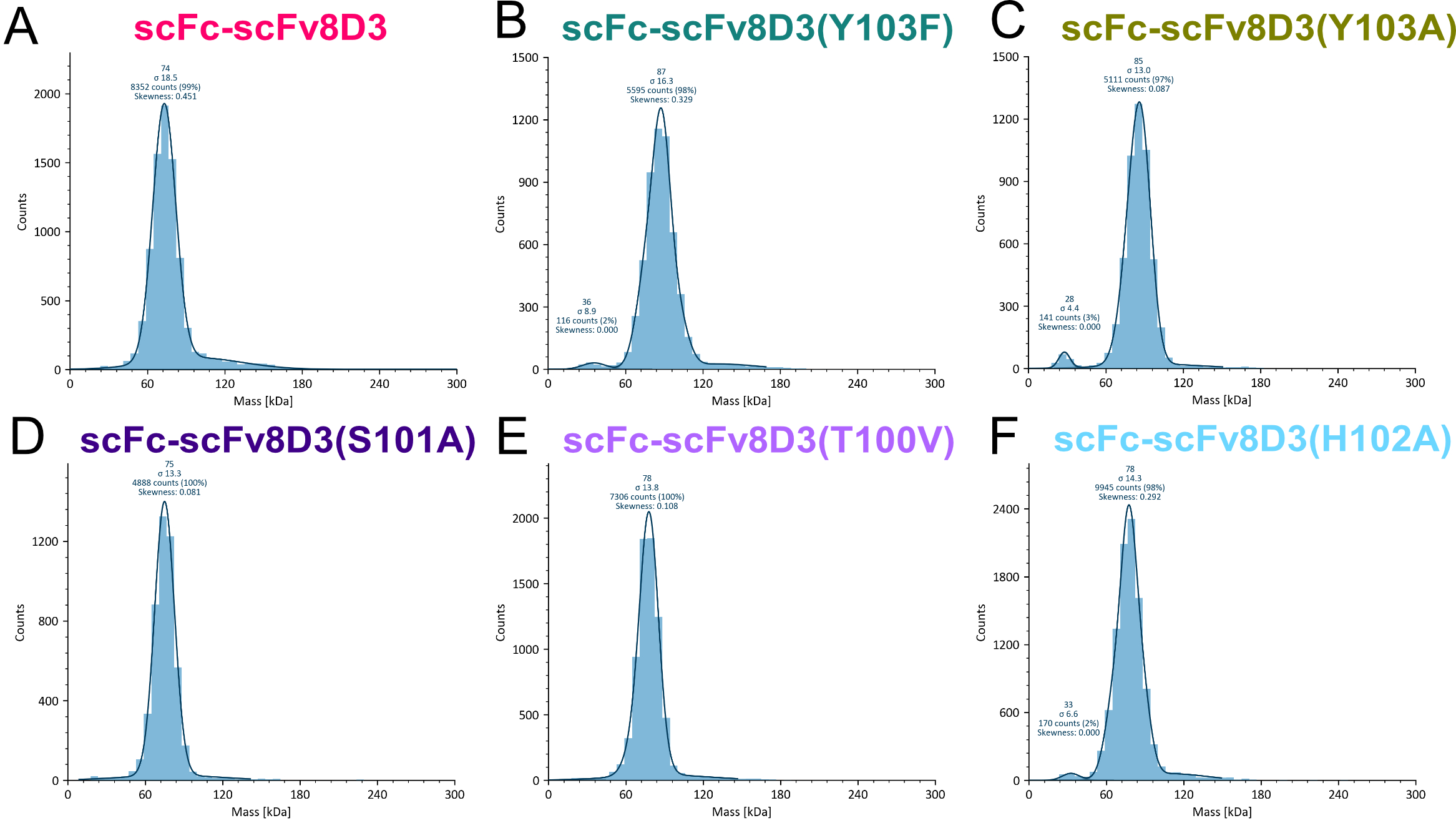


**Supplementary figure 4.** Mass photometry analysis of purified scFc-scFv8D3 and scFc-scFv8D3 affinity mutant constructs. (A) to (F) showed that they are monomeric. The measured molecular mass for each construct was close to the theoretical mass of 82 kDa. The very small peaks at approximately 30-40 kDa are likely minor buffer impurities.


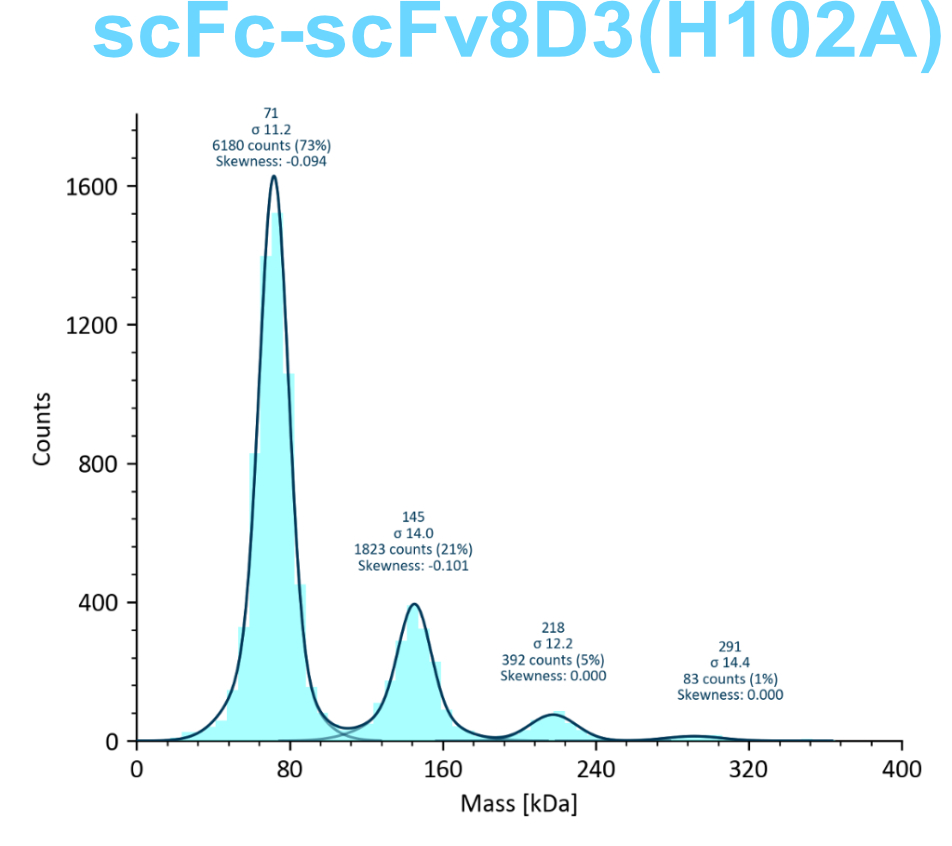


**Supplementary figure 5.** Mass photometry analysis of multimerized purified scFc-scFv8D3(H102A) affinity mutant construct showed multiple peaks pertaining to the monomeric form close to the theoretical mass of 82 kDa as well as dimeric form close to 164 kDa, trimeric form close to 240 kDa, and tetrameric form close to 320 kDa.

**
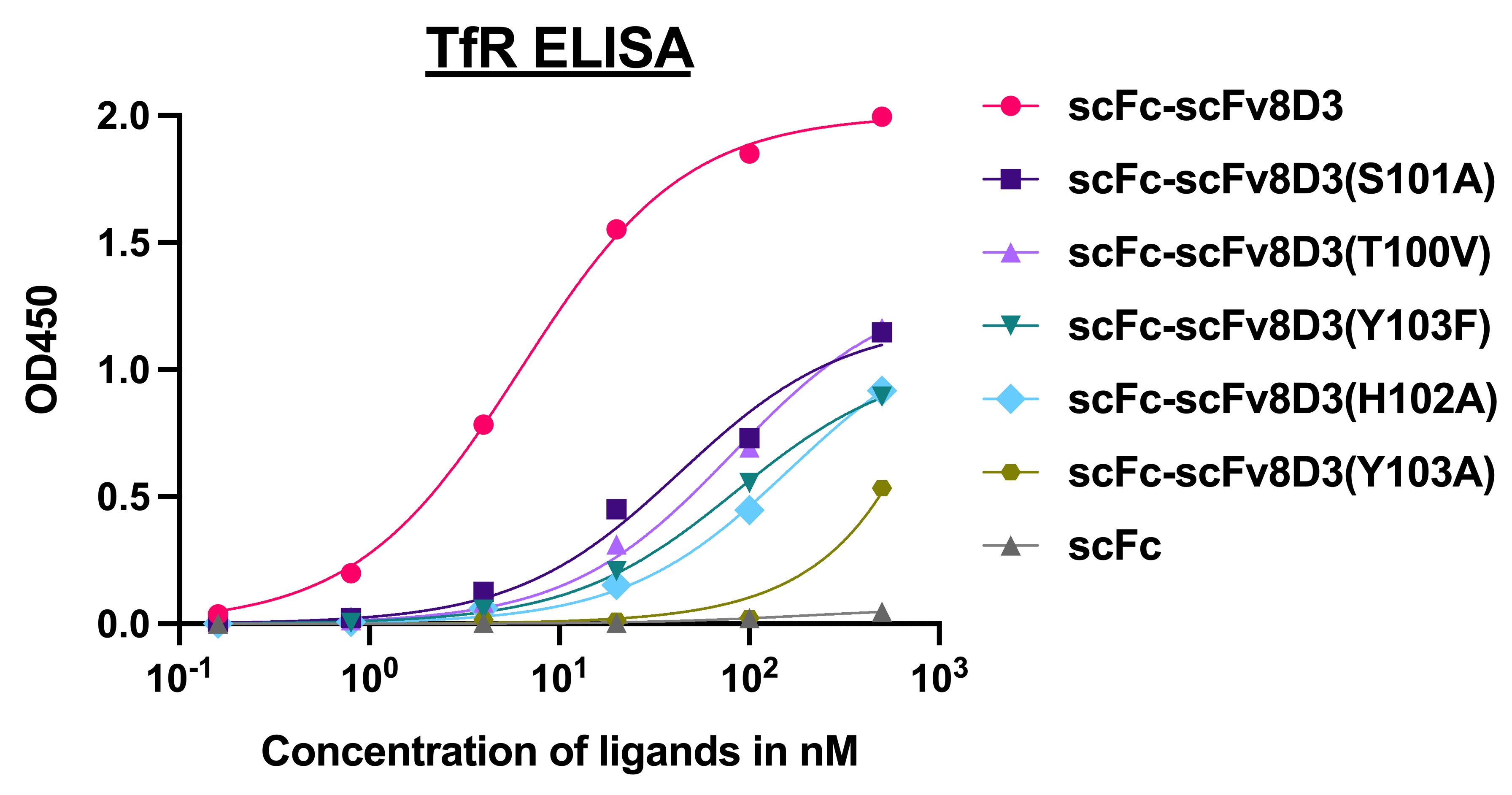
**

**Supplementary figure 6.** Non-normalizedTfR ELISA showing binding efficacy of scFc-scFv8D3 and affinity mutant constructs. Standard curves of each ligand were analyzed in duplicate and detected with an anti-IgG HRP-conjugated antibody recognizing Fc-region.


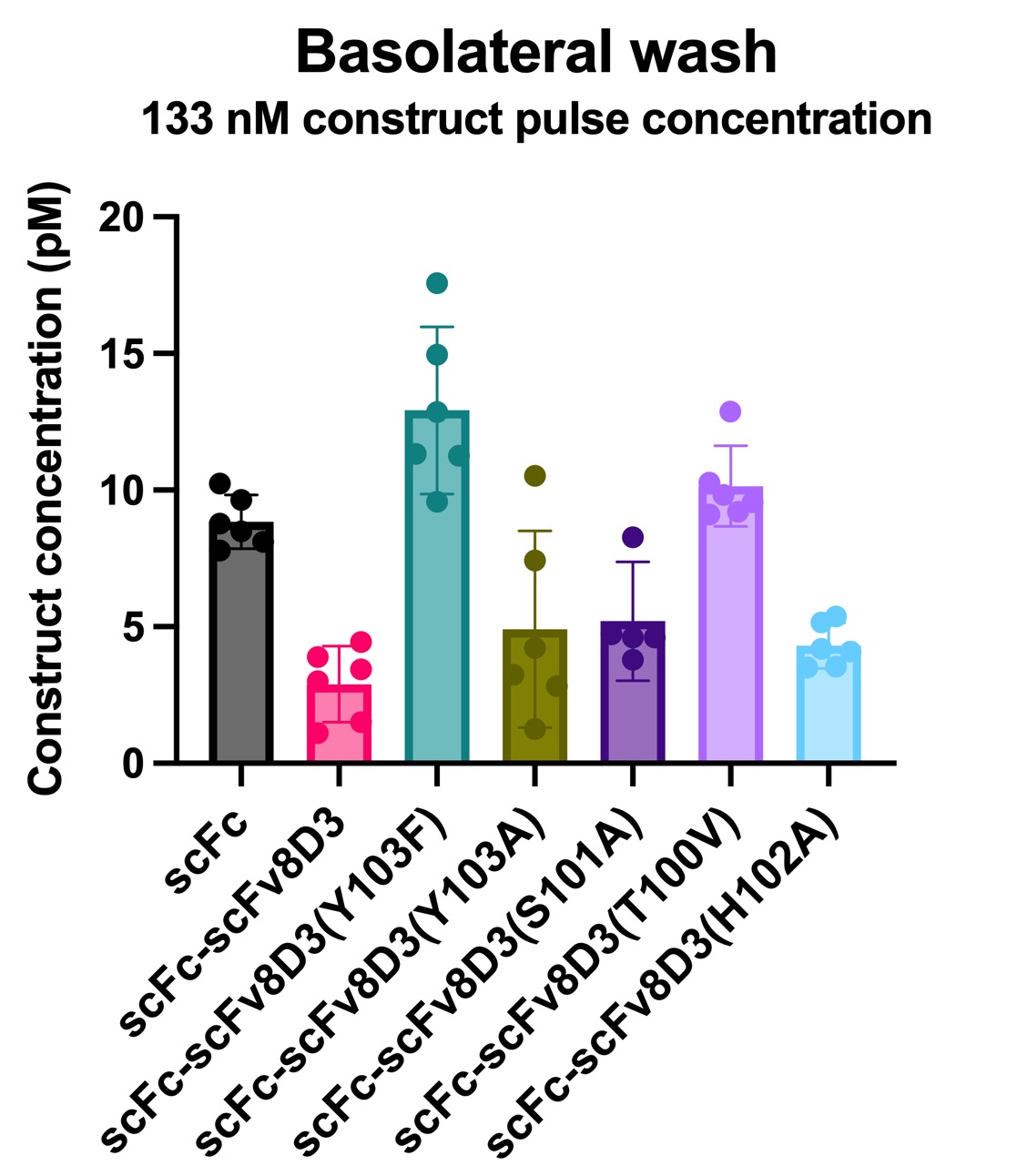


**Supplementary figure 7.** Basolateral wash of In-Cell BBB Trans assay. Graphical representation of average antibody concentrations found in the basolateral 6-hour chase wash of murine capillary endothelial cells plated on 24-well transwell cultures, following a one-hour “pulse” with 133 nM scFc, scFc-scFv8D3, scFc-scFv8D3(Y103F), scFc-scFv8D3(Y103A), scFc-scFv8D3(S101A), scFc-scFv8D3(T100V), and scFc-scFv8D3(H102A). Six transwells were used for each construct. The error bars represent 95% confidence intervals.

**Quality control TfR ELISA of ^125^I labeled affinity mutants**

**
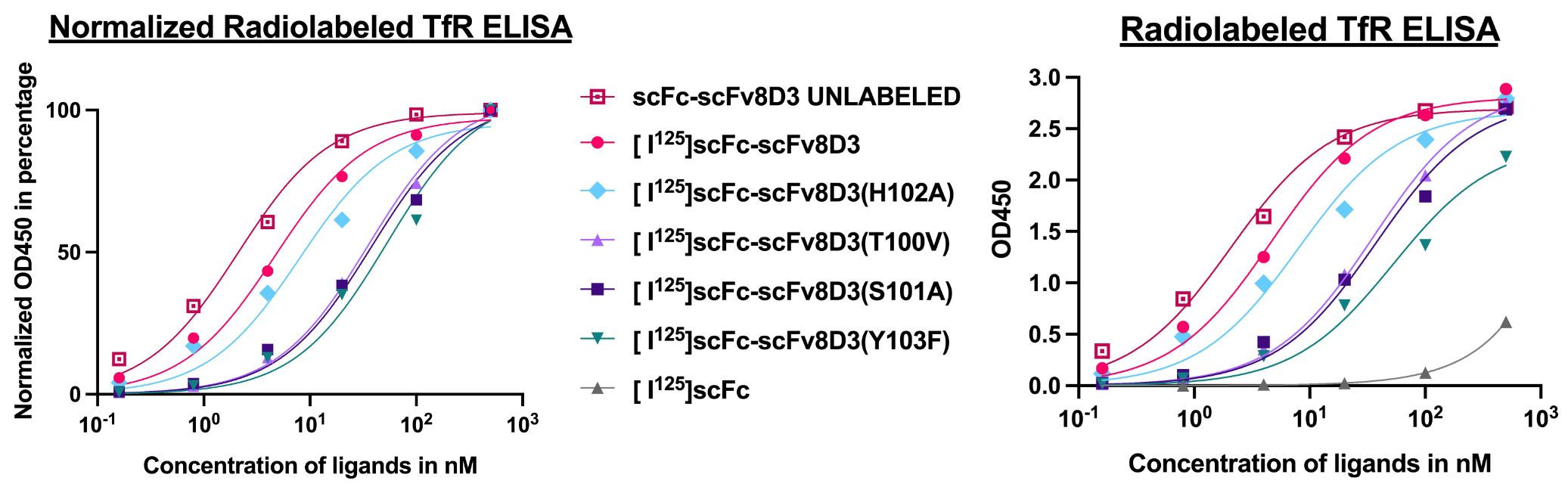
**

**Supplementary figure 8**. Quality control TfR ELISA showing binding efficacy of ^125^I-labeled scFc-scFv8D3 and affinity mutant constructs after concentration and radiolabeling for animal experiments. Standard curves of each ligand were analyzed in duplicate and detected with an anti-IgG HRP-conjugated antibody recognizing Fc-region. For the normalized graph (left) the binding curves were normalized to maximum binding signal of each construct. Non-linear regression curves were created using a “one site – specific binding” model.

**In vivo blood pharmacokinetics of ^125^I labeled affinity mutants including scFc-scFv8D3(H102A)**

**
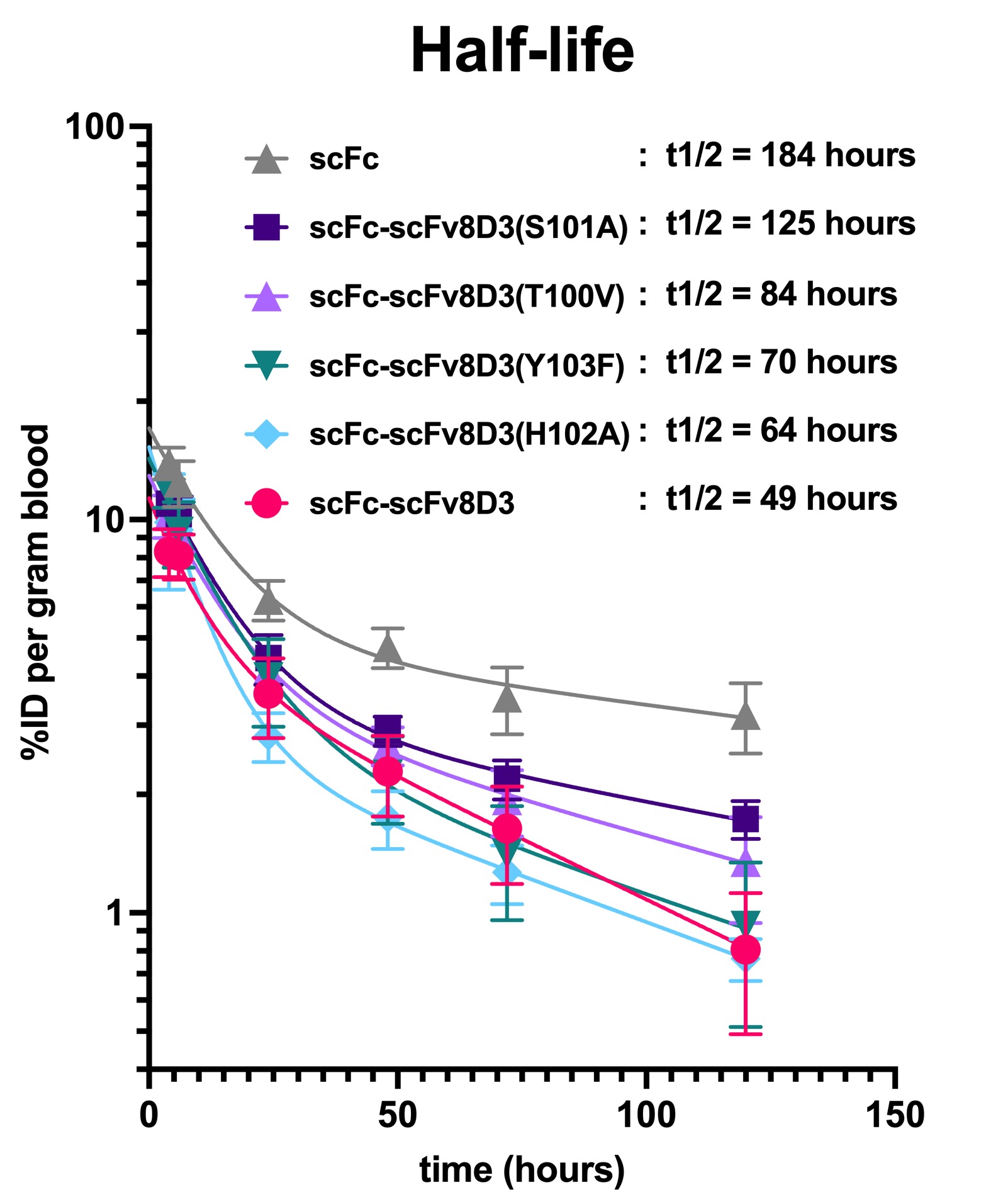
**

**Supplementary figure 9**. In vivo blood pharmacokinetics of ^125^I-labeled scFc-scFv8D3 and affinity mutant constructs in WT mice, including the scFc-scFv8D3(H102A) mutant construct. Blood concentrations expressed as a percentage of injected dose (%ID) per gram blood from blood samples taken at the indicated times, following intravenous injection of the ^125^I radiolabeled constructs at a therapeutic dose (30 nmol/kg). 30 nmol/kg corresponds to 2.5 mg/kg of scFc-scFv8D3 and the scFc-scFv8D3 affinity mutant constructs, while the same nanomolar concentration corresponds to 1.66 mg/kg for scFc. The half-life of each construct was calculated to be; 184 hours for scFc, 125 hours for scFc-scFv8D3(S101A), 84 hours for scFc-scFv8D3(T100V), 70 hours for scFc-scFv8D3(Y103F), 64 hours for scFc-scFv8D3(H102A), and 49 hours for scFc-scFv8D3.

Ex vivo brain concentration of ^125^I labeled affinity mutants including scFc-scFv8D3(H102A) 120 hours-post injection

At the end of the 120 hour blood-half life in vivo experiment, the gamma radiation levels in the brains of the mice were measured ex vivo to evaluate if the scFc-scFv8D3 affinity mutants also had increased brain concentration compared to scFc-scFv8D3 at this time point. There was very little of the scFv8D3-constructs left to detect in the brain at this late time point, which was expected as they all lack intra-brain targets, and therefore do not accumulate in the brain over time. In contrast to e.g. therapeutic antibodies targeting amyloid beta in AD mice. In addition, the signal measured at this time point is close to the detection limit of the method which lowers the credibility of the results. The brain concentrations are expressed as a percentage of injected dose (%ID) per gram of brain tissue. None of the mutants had increased brain concentration compared to the negative control scFc at 0.02 %ID/g.

**
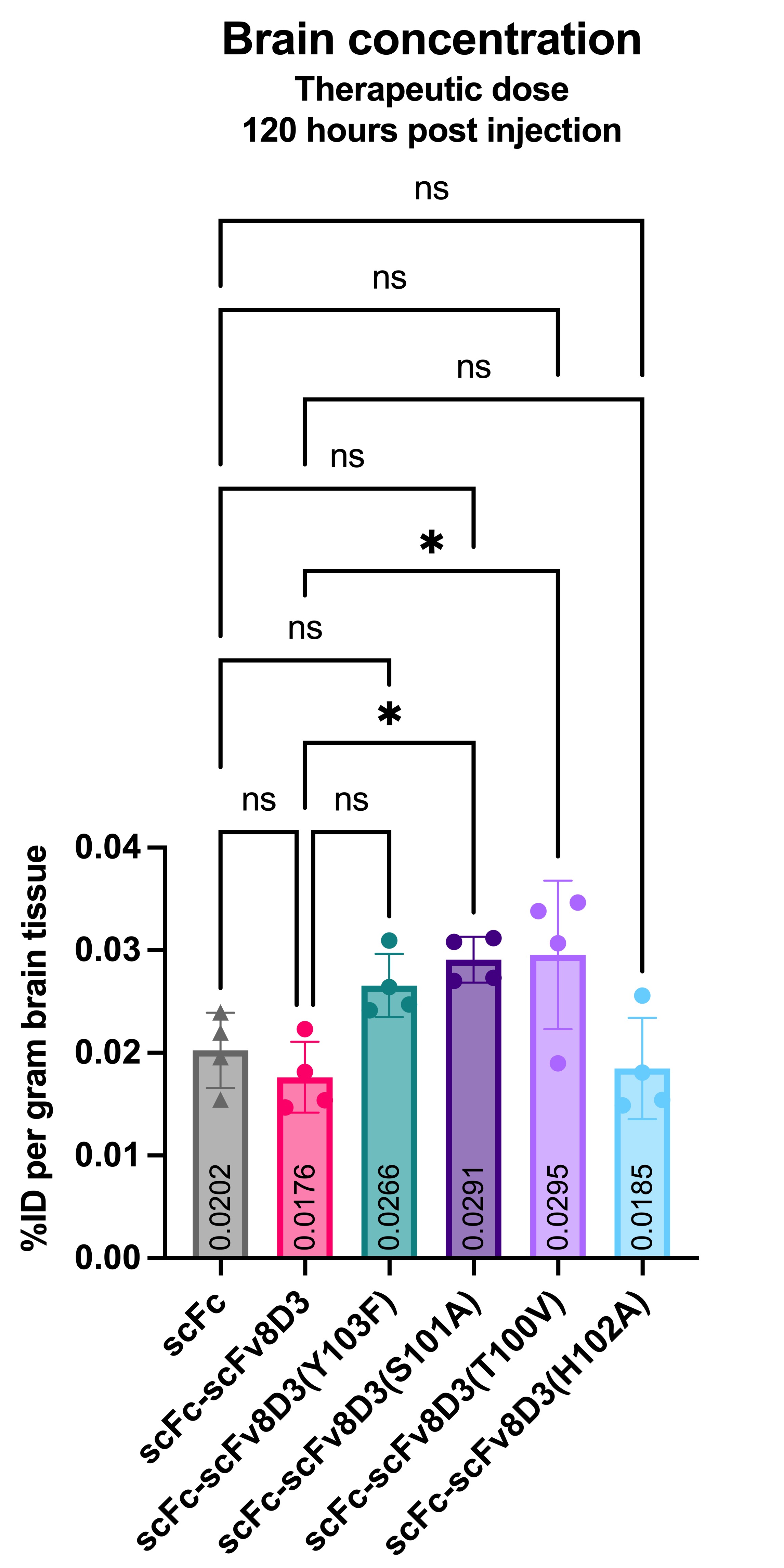
**

**Supplementary figure 10.** Brain concentration of ^125^I-labeled scFc-scFv8D3 and affinity mutant constructs in WT mice 120 hours post-injection. Brain uptake concentrations expressed as a percentage of injected dose (%ID) per gram of brain tissue from brains collected and measured for radioactivity ex vivo 120 hours post-injection, following intravenous injection of the ^125^I radiolabeled constructs at a therapeutic dose (30 nmol/kg). 30 nmol/kg corresponds to 2.5 mg/kg of scFc-scFv8D3 and the scFc-scFv8D3 affinity mutant constructs, while the same nanomolar concentration corresponds to 1.66 mg/kg for scFc. Results are presented as mean ±SD. Statistical pairwise comparisons were conducted between scFc-scFv8D3 and the scFc and scFc-scFv8D3 affinity mutant constructs. * Represents a significance P<0.05.

**Ex vivo brain concentration of ^125^I labeled affinity mutants including scFc-scFv8D3(H102A) 24 hours-post injection**

**
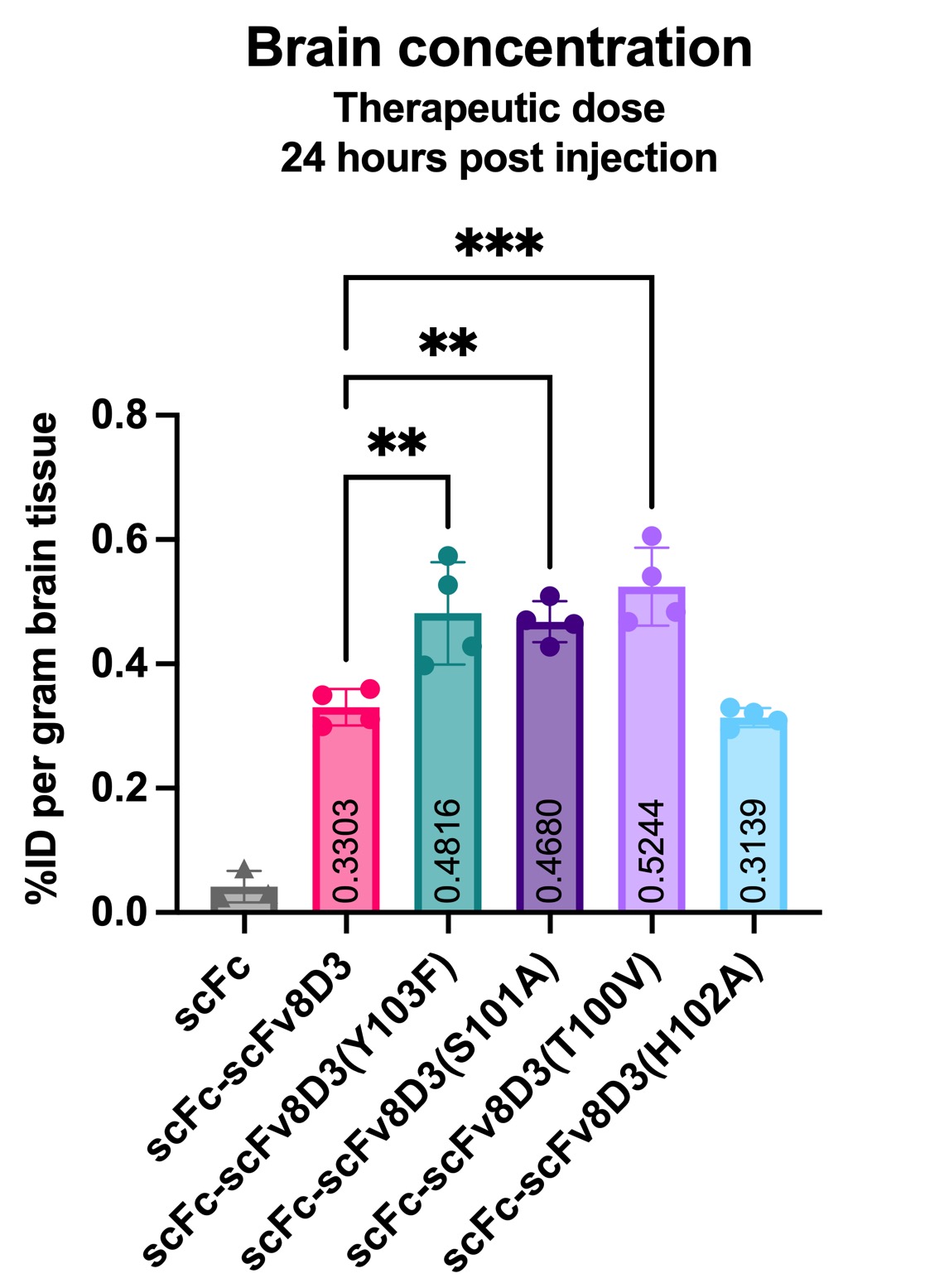
**

**Supplementary figure 11.** Brain concentration of ^125^I-labeled scFc-scFv8D3 and affinity mutant constructs in WT mice 24 hours post-injection. Brain uptake concentrations expressed as a percentage of injected dose (%ID) per gram of brain tissue from brains collected and measured for radioactivity ex vivo 24 hours post-injection, following intravenous injection of the ^125^I radiolabeled constructs at a therapeutic dose (30 nmol/kg). 30 nmol/kg corresponds to 2.5 mg/kg of scFc-scFv8D3 and the scFc-scFv8D3 affinity mutant constructs, while the same nanomolar concentration corresponds to 1.66 mg/kg for scFc. Results are presented as mean ±SD. Statistical pairwise comparisons were conducted between scFc-scFv8D3 and the scFc and scFc-scFv8D3 affinity mutant constructs. * Represents a significance P<0.05, ** represents P< 0.01, and *** represents P< 0.001.

**Peripheral biodistribution of scFc-scFv8D3 affinity mutants generally unaltered with the exception of blood distribution**

At the end of 120 hours in vivo experiment, the gamma radiation levels in other tissues of the mice were measured ex vivo to evaluate if the peripheral biodistribution of the scFc-scFv8D3 affinity mutants were altered compared to the biodistribution of scFc-scFv8D3. The biodistribution of the scFc-scFv8D3 affinity mutants were unaltered with the exception of distribution in blood and plasma (Supplementary figure 12). The scFc-scFv8D3(S101A) affinity mutant and the scFc-scFv8D3(T100V) affinity mutant were significantly increased in blood and in plasma compared to scFc-scFv8D (Supplementary figure 12), which reflect the longer blood half-time of the affinity mutants compared to scFc-scFv8D3 (Figure 7), and this pattern is mirrored by the scFc construct which is similarly significantly increased in blood and in plasma compared to scFc-scFv8D3 (Supplementary figure 12), while also having longer blood half-life than scFc-scFv8D3 (Figure 7).

At the end of the follow-up 24 hour ex vivo brain concentration experiment similar results were observed for the scFc-scFv8D3 affinity mutants compared to scFc-scFv8D3. The biodistribution was generally unaltered with the exception of increased plasma concentration for all of the mutants except scFc-scFv8D3(H102A) (Supplementary figure 13). In addition, increased blood concentration was observed for scFc-scFv8D3(S101A) and scFc-scFv8D3(T100V) and significantly increased concentration of scFc-scFv8D3(Y103F) and scFc-scFv8D3(S101A) was observed in bone (Supplementary figure 13).

**
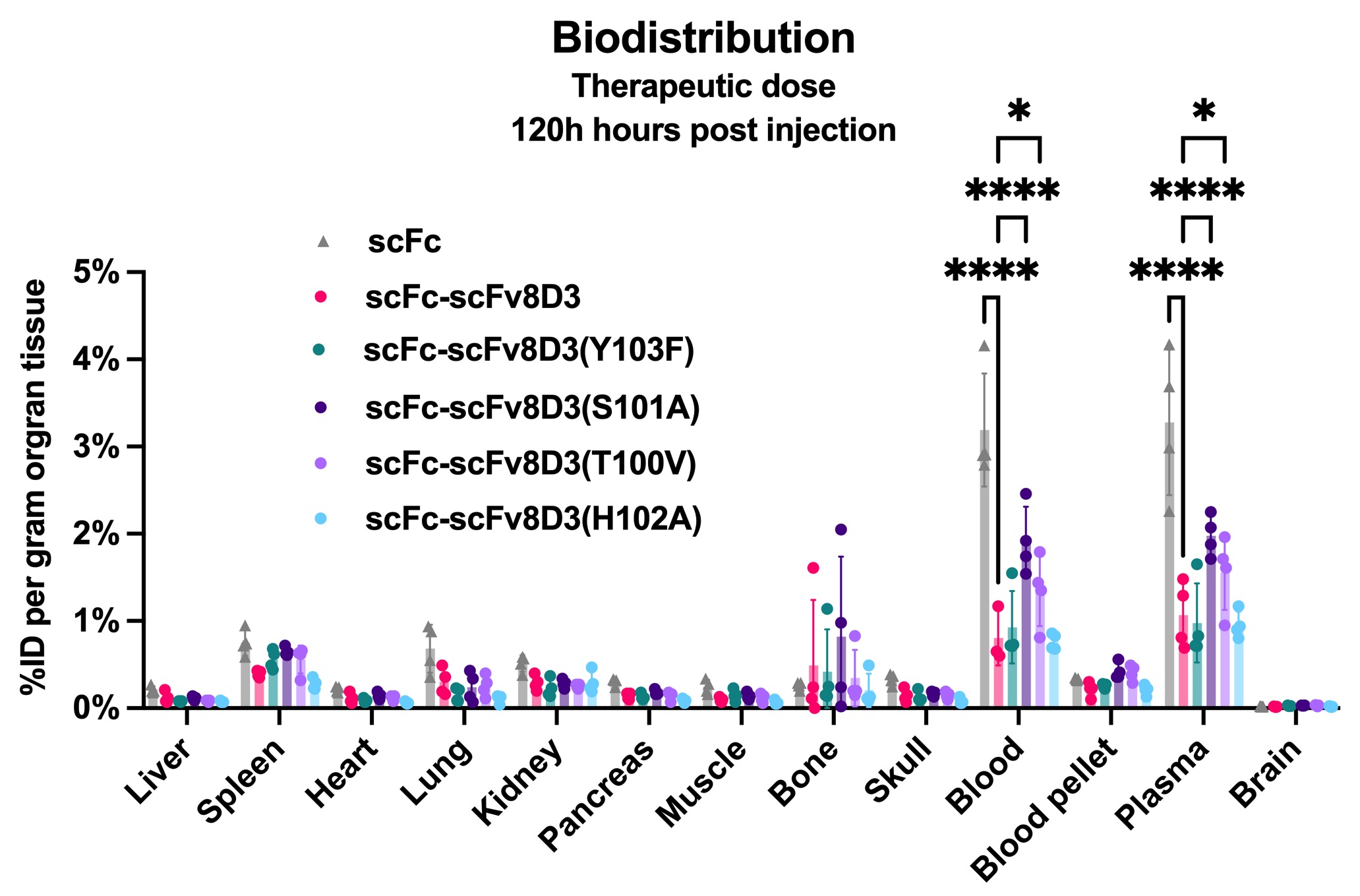
**

**Supplementary figure 12.** Biodistribution of scFc-scFv8D3 and scFc-scFv8D3 affinity mutants. Biodistribution expressed as percentage of injected dose (%ID) per gram tissue from tissue samples measured for radioactivity ex vivo 120 hours post-injection, following intravenous injection of the ^125^I radiolabeled constructs at a therapeutic dose (30 nmol/kg). 30 nmol/kg corresponds to 2.5 mg/kg of scFc-scFv8D3 and the scFc-scFv8D3 affinity mutant constructs, while the same nanomolar concentration corresponds to 1.66 mg/kg for scFc. Results are presented as mean ±SD. Statistical pairwise comparisons were conducted between scFc-scFv8D3 and the scFc and scFc-scFv8D3 affinity mutant constructs. * Represents a significance P<0.05, **** represents P< 0.001.


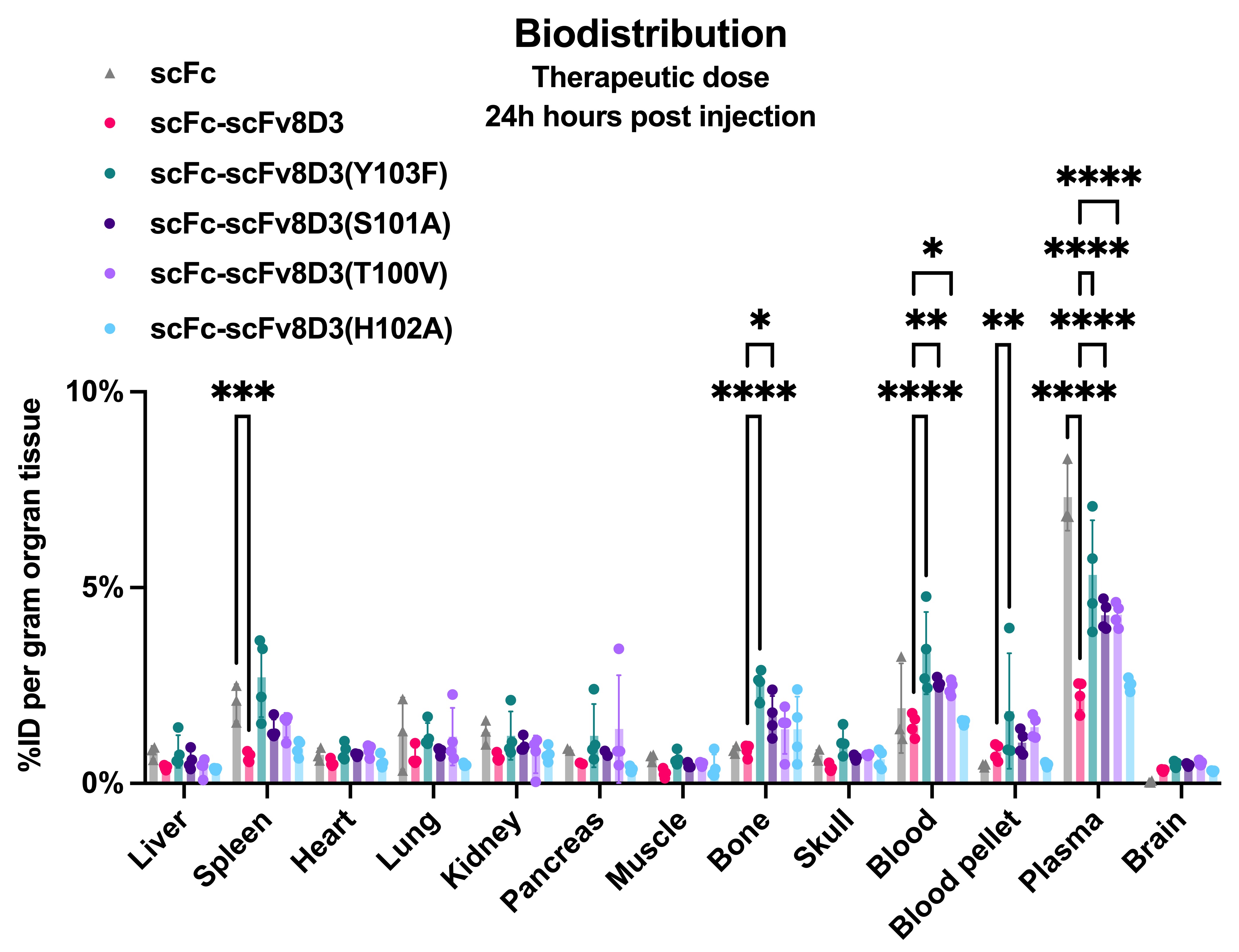


**Supplementary figure 13.** Biodistribution of scFc-scFv8D3 and scFc-scFv8D3 affinity mutants. Biodistribution expressed as percentage of injected dose (%ID) per gram tissue from tissue samples measured for radioactivity ex vivo 24 hours post-injection, following intravenous injection of the ^125^I radiolabeled constructs at a therapeutic dose (30 nmol/kg). 30 nmol/kg corresponds to 2.5 mg/kg of scFc-scFv8D3 and the scFc-scFv8D3 affinity mutant constructs, while the same nanomolar concentration corresponds to 1.66 mg/kg for scFc. Results are presented as mean ±SD. Statistical pairwise comparisons were conducted between scFc-scFv8D3 and the scFc and scFc-scFv8D3 affinity mutant constructs. * Represents a significance P<0.05, **** represents P< 0.001.
